# MONGKIE: an integrated tool for network analysis and visualization for multi-omics data

**DOI:** 10.1186/s13062-016-0112-y

**Published:** 2016-03-18

**Authors:** Yeongjun Jang, Namhee Yu, Jihae Seo, Sun Kim, Sanghyuk Lee

**Affiliations:** Ewha Research Center for Systems Biology (ERCSB), Ewha Womans University, Seoul, Republic of Korea; Interdisciplinary Program in Bioinformatics, College of Natural Science, Seoul National University, Seoul, Republic of Korea

**Keywords:** Network visualization, Network modeling, Graph clustering, Omics data analysis, Over-representation analysis

## Abstract

**Background:**

Network-based integrative analysis is a powerful technique for extracting biological insights from multilayered omics data such as somatic mutations, copy number variations, and gene expression data. However, integrated analysis of multi-omics data is quite complicated and can hardly be done in an automated way. Thus, a powerful interactive visual mining tool supporting diverse analysis algorithms for identification of driver genes and regulatory modules is much needed.

**Results:**

Here, we present a software platform that integrates network visualization with omics data analysis tools seamlessly. The visualization unit supports various options for displaying multi-omics data as well as unique network models for describing sophisticated biological networks such as complex biomolecular reactions. In addition, we implemented diverse in-house algorithms for network analysis including network clustering and over-representation analysis. Novel functions include facile definition and optimized visualization of subgroups, comparison of a series of data sets in an identical network by data-to-visual mapping and subsequent overlaying function, and management of custom interaction networks. Utility of MONGKIE for network-based visual data mining of multi-omics data was demonstrated by analysis of the TCGA glioblastoma data. MONGKIE was developed in Java based on the NetBeans plugin architecture, thus being OS-independent with intrinsic support of module extension by third-party developers.

**Conclusion:**

We believe that MONGKIE would be a valuable addition to network analysis software by supporting many unique features and visualization options, especially for analysing multi-omics data sets in cancer and other diseases.

**Reviewers:**

This article was reviewed by Prof. Limsoon Wong, Prof. Soojin Yi, and Prof. David Kreil.

**Electronic supplementary material:**

The online version of this article (doi:10.1186/s13062-016-0112-y) contains supplementary material, which is available to authorized users.

## Implementation

### Introduction

Given the huge volume and complexity of omics data such as those from TCGA (The Cancer Genome Atlas) projects, it is a major challenge to gain insights into biological principles [1]. A commonly used powerful approach in addressing such challenge is to use the network-based integrative analysis which requires insightful visualization in addition to network analysis tools [2]. Cytoscape has been widely and effectively used for visualizing biological networks with ample plugin programs for analysis [3]. However, integration and utility of third-party plugins are rather limited, not satisfying some important requirements such as dissecting the complex network into multiple small parts of functionally or topologically related nodes and subsequent visualization of each module in distinct styles [4]. Visual comparison of multiple experiments on the network often requires tedious procedure due to the lack of overlaying functions [5]. Another important limitation is that most network programs support binary interactions only and it is difficult to describe complex biochemical reactions or pathway interactions. Many of above features are not easily accomplished as plugin applications of Cytoscape without modifying the source code of core engine. Thus, a new platform for visual data mining of biological networks is much needed.

MONGKIE (**Mo**dular **N**etwork **G**eneration and Visualization with **K**nowledge **I**ntegration **E**nvironments) was developed as a general platform for interactive visualization and analysis of complex omics data in the context of biological networks. Several algorithms for network analysis were implemented and they are tightly coupled with the visualization tools in a unified platform.

### Functionalities

Like other network tools, MONGKIE supports basic graph representations (4.1 in Additional file 1) and diverse interactive ways to explore or edit a network including the visual editor, data-to-visual mapping (4.3 in Additional file 1), zooming, filtering, searching (4.4 in Additional file 1), and various graph layouts (4.5 in Additional file 1) etc. A unique feature is the extension of network model beyond the basic graph representation of binary interactions to support multi-modal relationships and hyper-graphs [2]. This extension allows us to describe complex, interwoven biochemical reactions such as formation of protein complexes or pathway reactions controlled by other components (4.2 in Additional file 1).

**Fig. 1:**
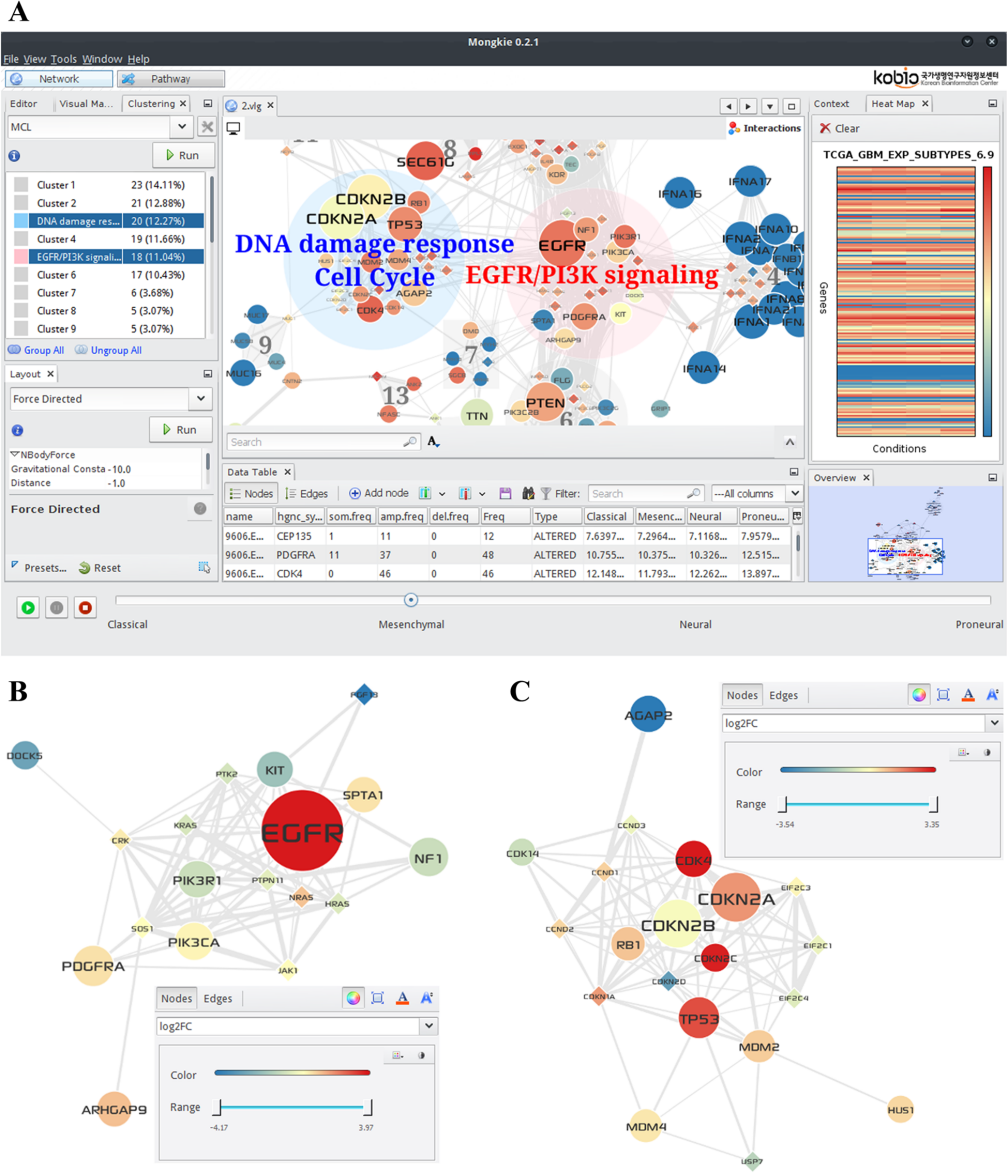
Screenshots from MONGKIE for GBM-altered network and core gene modules. **a** User interface for network clustering (*top left*), visualization of a network and clusters (*center*), and expression levels in 4 GBM subtypes (*top right*). **b** EGFR-PI3K signaling module. This module is defined as a group node in the main network (shown in *pink circle* in **a**). **c** DNA damage response and cell cycle module (group node in *blue circle*). Altered and linker genes are represented by circle and diamond nodes, respectively. The alteration frequency and expression correlation were mapped to the node size and the edge width, respectively. The node color shows the average gene expression in GBM patients of mesenchymal subtypes (**a**) and log_2_(FoldChange) between tumor vs. normal condition in all GBM patients (**b** and **c**). In **a**, patient groups can be switched manually in the *bottom panel* or automatically to show animated pictures

Another distinguishing feature is to support creation and manipulation of network subgroups. Each subgroup can be laid out separately from other modules in distinct styles (Fig. 1a and Fig. 3.2 in Additional file 1). Such geometric separation is critical to focus on particular modules of interest without being disturbed by unnecessary interactions (5.1 in Additional file 1). For example, users can easily examine pathways affected by mutated genes or cross-talks between functional modules in cancer.

MONGKIE provides various methods to define subgroups or network modules. Several network clustering algorithms are implemented to facilitate identification of densely interconnected network modules whose components are functionally related. Each cluster can be defined as a group, member genes of which are gathered for facile visualization (Fig. 1a). Such modular organization of complex network is often valuable in obtaining biological insights and deducing molecular mechanisms. To aid functional interpretation of network modules, we provide a utility to examine statistical enrichment of Gene Ontology terms (5.3 in Additional file 1). All components for visualization, network analysis for defining subgroups, and functional interpretation of network modules can be easily threaded into a pipeline that allows user interaction at each step.

Another useful feature is overlaying experimental data (e.g. gene expression) onto the network space using the data-to-visual mapping function. We further implemented serial (manual) or animated visualization of multiple experimental profiles on an identical network with a separate window for a heat-map view (Fig. 1a). This feature facilitates exploring complex data under various conditions (e.g. different subtypes of cancer or time series data) to identify co-regulatory patterns within the network components (5.2 in Additional file 1). Cytoscape has a similar function in the VistaClara plugin [6] program, but it is available in the old legacy version (Ver.2.8) only.

### User case study

To demonstrate the utility of MONGKIE as general network analysis and visualization software, we analyzed multi-omics data from the TCGA Glioblastoma Multiforme (GBM) consortium [7]. Our goal was to identify driver gene candidates among mutated genes and core regulatory modules with functional interpretation.

From the TCGA GBM data sets (3.1 in Additional file 1), we manually selected recurrently altered genes with somatic mutations (patient freq. > 0.02) or copy number aberrations (patient freq. > 0.03) as the starting gene set. The GBM-altered network was established by extracting all shortest paths among each pair of altered genes in the STRING network (score > 900) [8] with distance threshold 2 and then retaining significant linkers only (*p*-value < 0.01) [9] as described in 3.2 in Additional file 1. Then, we assigned the Pearson correlation coefficient of expression levels in tumor samples as the edge weight between two genes. This custom network was imported into MONGKIE as a background network using the Interaction Manager function. Users may explore or modify the network at this stage.

Next, we applied the MCL clustering algorithm [10] to identify network modules, representing genes with correlated expression and topological proximity (3.4 in Additional file 1). The grouping utility of MONGKIE allows users to illustrate the network modules and their member genes simultaneously on top of background network as shown in Fig. 1a. This unique feature helps users examine biological roles of network subgroups by the optimized layout and subsequent GO enrichment analysis.

Among the top five largest modules that we discovered, two were in agreement with previously known GBM signaling networks [7, 11] (3.5 in Additional file 1): (i) *EGFR*-*PI3K* signaling including *EGFR*, *PDGFRA*, *PIK3CA* and *PIK3R1* (Fig. 1b), and (ii) DNA damage response and cell cycle regulation including *TP53*, *CDKN2A*/*B*, *CDK4*, *MDM2*/*4* and *RB1* (Fig. 1c). Gene sets in 2 critical modules and their functional annotations are provided in Table 3.2 in Additional file 1. The result demonstrates that cancer driver genes and core gene modules can be identified and effectively visualized in MONGKIE with all necessary features already implemented for user convenience.

Compared to the NetBox program [9] where the background network was fixed as their own global Human Interaction Network, MONGKIE offers greater flexibility in choosing background network and clustering algorithms. Furthermore, our example of importing customized networks, clustering, visualization of identified clusters with group function, and functional annotation with GO enrichment analysis can be easily setup as a pipeline for routine usage.

### Software architecture

**Fig. 2:**
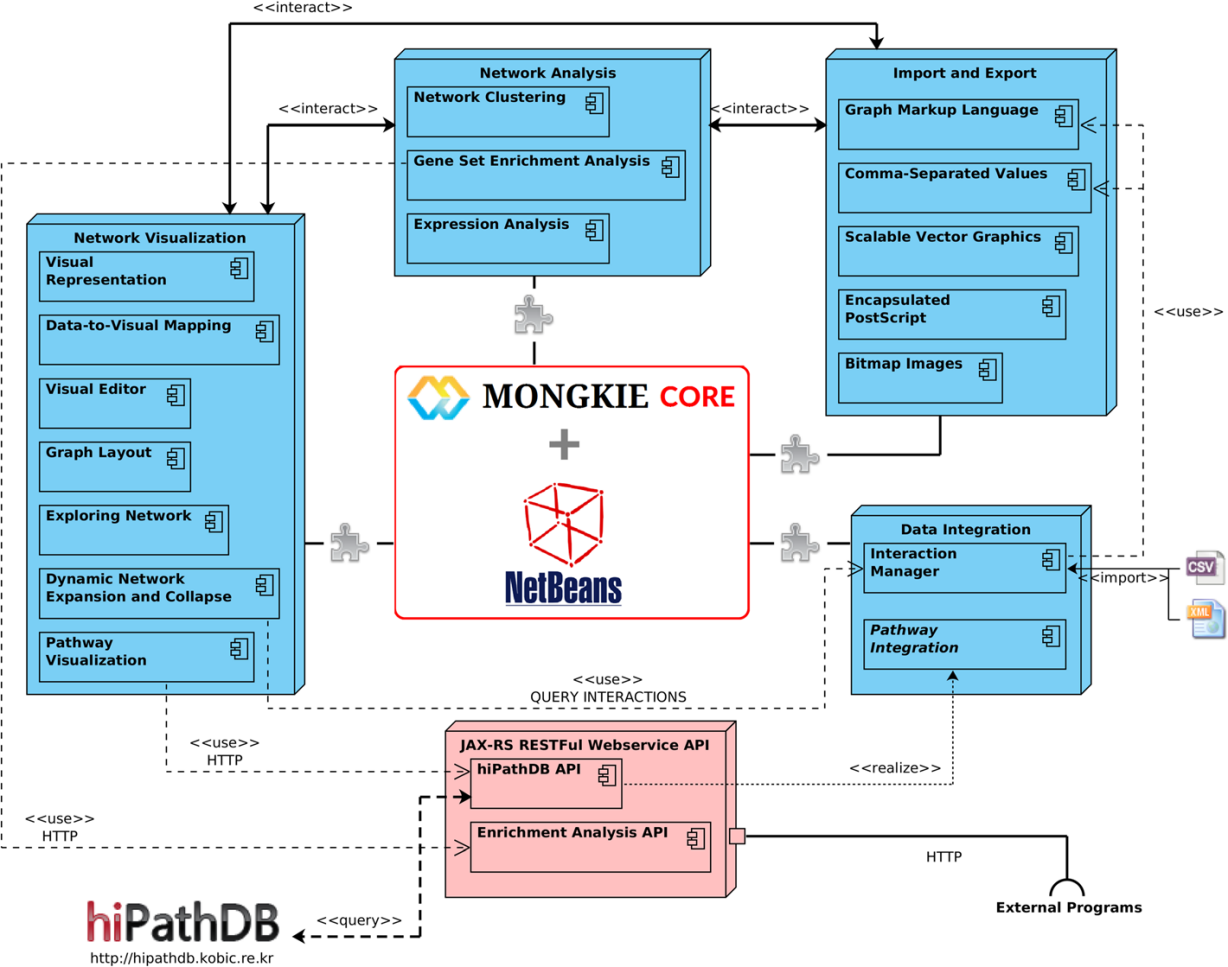
Overview of software architecture implemented in MONGKIE. The *blue blocks* represent the core functional parts of the platform such as graph visualization, network analysis, data integration, import and export. The *pink block* represents the remote web service APIs that could provide data or analysis as requested by external programs. Boxes in each functional part are plugins pre-implemented using the APIs, SPIs, and UI components of MONGKIE. Each plugin can expose its own APIs so that other plugin programs can utilize them. This makes it possible to develop plugins for a plugin. For example, we implemented the MCL algorithm as a plugin application of the network clustering plugin

MONGKIE was built on top of the NetBeans RCP (Rich Client Platform) [12] that supports the plugin architecture, thus it is easy to implement various new plugins with additional functionalities. A schematic overview of its plugin architecture is given in Fig. 2.

Based on its extensible architecture, MONGKIE provides well-defined APIs (Application Programming Interface), SPIs (Service Provider Interface), and UI (User Interface) widgets for the base functionalities, including graph visualization and network analysis. This allows plugin developers to focus on novel algorithms and features without being concerned about auxiliary tasks such as UI components, event handling, and the visualization scheme. These fundamental functions are provided as the black-box features, allowing a great deal of flexibility in building various improvements of existing plugins as well as introducing new functionalities.

MONGKIE also provides the *Plugin Manager*, shown in Fig. 9.2B in Additional file 1, so that users can install, update, remove, activate, or deactivate individual plugins. Thus, users can customize the application functionalities according to their needs.

Of note, the JAX-RS (The Java API for RESTful Web Services) [13] technology was applied in MONGKIE for serving remote data and analyses in a unified way. Based on these web APIs, we provide the functionality that allows users to access our integrated data sources or analysis services not only within the MONGKIE platform but also from any client programs supporting the REST (Representational State Transfer) connection (Fig. 9.3 in Additional file 1). For example, we provide the RESTful web services API for the hiPathDB [14] database and the gene ontology (GO) over-representation analysis (Fig. 2).

In addition, MONGKIE was built to support multi-tasks that allow users to run several tasks simultaneously in separate threads without blocking user interactions with the program. For example, you can load the gene expression profiles onto the network while running a layout algorithm on the same network.

### Comparison with Cytoscape

Cytoscape is the leading platform for network visualization and analysis. Being a community standard, it supports diverse algorithms and visualization schemes provided by core and third-party developers. Even though the utilities of MONGKIE are rather limited currently, we tried to implement most of the essential functions in network analysis and visualization tools so that users do not experience inconvenience in typical applications. Here, we highlight the distinguishing features of MONGKIE in comparison with those of Cytoscape.

#### Interaction source management

A typical way of generating a molecular network in Cytoscape is to define a subset of genes and then build a network using public interaction databases through the plugin specific for each data source or through the PSICQUIC [15] universal client. However, it is often the case that users want to work on their own networks that are context-specific to the biological problems of interest. Then, users have to set up an API server to interrogate the customized network, which is a cumbersome procedure to most users. The GBM-altered network in the user case study represents an example of customized network that were obtained by a series of computational steps starting from the STRING interaction database.

The *Interaction Manager* in MONGKIE allows users to import a (large-sized) customized network as the background network model without developing any programming interface or web server (6.2 in Additional file 1). Starting from the genes of interest, users can then expand the network successively through the custom network for exploring and data mining purposes. This feature is especially useful for analyzing large-sized networks where the complete network is difficult to handle as a whole. Additionally, the imported custom network is locally available on the next run with no need of importing again. The procedure for working with the custom network is illustrated in Fig. 6.1B in Additional file 1.

#### Group functionalities

Group nodes in MONGKIE are a kind of hyper-nodes that contain multiple nodes. We provide various ways of defining group nodes. For example, group nodes can be defined as collections of densely connected nodes through network clustering or as collections of nodes with the same function after GO over-representation analysis. Manual selection of group members is possible as well. The visual style (e.g. shape, color, font) of the group nodes can be customized in the visual editor window (Fig. 4.3C in Additional file 1). Importantly, we modified the force-directed layout algorithm [16] so that nodes within the same group attract each other and nodes in different groups repel each other. Thus, group nodes are naturally positioned separately from other groups, minimizing overlapping regions among groups. Group nodes can then be manipulated just like general nodes for any exploratory purposes. On the other hand, Cytoscape’s group is a simple collection of nodes without dedicated manipulation or visualization scheme. The RBVI plugins [17], available only in the legacy version (Ver.2.8), were developed to augment the Cytoscape’ group management and visualization, but are still much different from MONGKIE’s group features.

#### Layout animation and dynamic configuration

Unlike Cytoscape, we use the force-directed layout algorithm as the default because it usually produces well-organized networks by minimizing edge crossings and recalculating attractive and repulsive forces iteratively [16]. The process of optimizing network layout is shown in animation and users may stop iteration once a good layout is obtained. Furthermore, we support the dynamic configuration in the layout process, where any changes in the layout settings are fed into the layout output even while the algorithm is running. The example of configuring settings for the force-directed layout is shown in Fig. 4.6 of Additional file 1.

#### UI flexibilities

In Cytoscape, the main application windows are made up of possibly multiple instances of network views in the center along with 4 panels classified according to application functions. Each panel is floatable or dockable with application tabs tightly attached to the panel. This UI offers a rather limited flexibility in window management. MONGKIE’s UI, based on the NetBeans docking framework, provides a fully flexible window system that supports many additional features such as detachable application tabs, auto-hide and slid window function on mouse-over, and drag-and-drop to any positions in the screen. Users may create a custom UI optimized for specific purposes or data types, and the custom window settings can be applied to the following sessions.

## Availability and requirements

**Project name:** MONGKIE

**Project home page:**http://yjjang.github.io/mongkie

**Operating system(s):** Platform independent

**Programming language:** Java

**Other requirements:** Java 7 or 8

**License:** GNU AGPL version 3

**Any restrictions to use by non-academics:** restricted by the license

## Reviewers’ comments

### Reviewer 1: Dr. Limsoon Wong

**Summary and Recommendations**: This manuscript describes a flexible GUI for visualizing and analyzing omics data in the context of biological network. The GUI looks good to me. However, the comparison with Cytoscape seems superficial and overly one sided.

**Authors’ response***: We thank Prof. Wong for the positive judgement on our GUI. We have added more descriptions on the unique features and comments on Cytoscape. Please see the response to the comments from other reviewers as well.*

**On revision:** I agree that MONGKIE has some GUI new features that Cytoscape does not have. However, impact of these features on visual analysis of biological networks (and the superiority over Cytoscape) is not very clearly demonstrated. Nonetheless, I am satisfied with this revised manuscript.

**Authors’ response**: *We thank Prof. Wong for the positive judgement on the revision.*

### Reviewer 2: Dr. Soojin Yi

**Summary**: Network analyses of multiple omics data require efficient and informative visualization. The authors present a new platform named MONGKIE. This tool represents an improvement over previous platforms in the following aspects: by supporting multi-modal hyper graphs, offering easy means to define subgroups, as well as to integrate with empirical data visualization. Considering that large scale data sets are generated in nearly daily basis, robust visualization/analyses tools such as MONGKIE are in high demand. I consider this tool to be an excellent contribution to fill the growing need of the community.

**Authors’ response**: *We thank Prof. Yi for the succinct and clear summary of our work as well as the encouraging appraisal.*

**Recommendations**: I have several minor comments. In the description of the case study (last paragraph/first paragraph of p.5-6), the authors mention that there were five large modules they identified, and mentioned that two were in agreement with previously known modules. What is the reference the authors are referring to? And also, what are their opinions about the other modules that were discovered?

**Authors’ response**: *We have added references for genetic and pathway alterations known in GBM pathogenesis. The other three modules also seem be relevant to GBM biology as well although they need more concrete experimental validations. The cluster 1 included angiogenesis-related genes such as KDR encoding one of the two receptors of VEGF* [18] *and TEK receptor tyrosine kinase* [19]*. The cluster 2 comprised DCTN2 and TUBGCP2 genes that are located in centrosome and related to the DNA damage repair signaling* [20]*. The cluster 4 included several genes from IFNA family that are proximal to CDKN2A on chromosome 9p. Since the focal deletion of CDKN2A is a well-known factor in GBM tumorigenesis* [9]*, the cluster 4 may represent the passenger alterations from the focal deletion.*

One option that the authors might include (which may already exist but I couldn’t quite figure out how to access, if there is any) is generating automatic log of all the functions that are being executed.

**Authors’ response**: *We appreciate a valuable suggestion. In the latest release (version 0.2.1), we have included a feature showing logs from the main system functions. Users can open the log window, by clicking ‘Window’-‘Output window’ in the menu bar or by pressing F4. The system logs (*e.g. *startup events, memory usage,* etc.*) are accessible by clicking ‘View’-‘IDE log’ in the menu bar.*

It would be nice if the comparison to Cytoscape had direct mention of the case study data analyses.

**Authors’ response**: *We modified the user case study accordingly. Comparison to Cytoscape was substantially improved as requested by Prof. Kreil (see below).*

### Reviewer 3: Dr. David Kreil

**Summary**: An integrated analysis of multiple data types is becoming a natural and crucial component in analysis of biological systems [Searls et al., 2005]. The incorporation of multiple sources of evidence into analyses has been shown to be of benefit in the modelling of complex molecular interactions [e.g., Hartemink et al., 2002; Nariai et al., 2005]. Specifically, network approaches could successfully contribute to a better understanding of biological processes and their role in diseases [Barabási 2007, Silverman & Loscalzo, 2012, 2013]. In the present manuscript, the authors introduce MONGKIE - a new integrative framework for the analysis and visualization of multi-omics networks. Well established tools for network analysis such as Cytoscape already support interactive network exploration and visualization. Cytoscape, moreover, implements a flexible plugin extension system. Some of the plugins also allow more advanced tasks, like exploring data with sub-structure or longitudinal dependencies, and the identification of co-regulatory patterns in networks. With Cytoscape widely used and providing a flexible environment for further development, it would therefore be natural to extend this framework, e.g., by building plugins for Cytoscape instead of building an entirely new tool. The authors do discuss their tool in comparison to Cytoscape to justify the development of a novel system: They argue that the paradigms underlying Cytoscape, even though sufficiently flexible in most analysis scenarios, are missing some functionalities essential for their work, and which cannot easily be provided within the Cytoscape plugin architecture.

**Authors’ response**: *We thank Prof. Kreil for careful evaluation of our work. Our responses to critical comments can be found below.*

Disappointingly, however, they support their claims just by a single “User case study”. As a key example feature, the authors there discuss the use of customized network annotations versus using network annotations from public repositories. It is, however, not at all clear how this is any different from using imported custom networks in Cytoscape [see: http://wiki.cytoscape.org/Cytoscape_User_Manual/Creating_Networks].

**Authors’ response**: *We acknowledge that our description on using customized networks was not clear in terms of difference with Cytoscape. To resolve the confusion, we completely rewrote the relevant section (‘Interaction source management’). As described in the suggested link, Cytoscape supports 4 different ways of creating networks as provided in suggested link: (1–2) importing pre-existing, formatted or unformatted network files, (3) importing networks from web services, (4) creating an empty network and manually adding nodes and edges. The main difference is related to (3) importing networks from web services. Whereas Cytoscape allows users to access remote data sources via web service clients of public repositories or pre-implemented plugins, the customized network in MONGKIE is imported* via *the ‘Interaction Manager’ and stored locally. The imported network serves as the background network model just like the web service clients in Cytoscape. Then, users can utilize the custom network for any exploratory purposes (*e.g. *such as extending neighbors, querying interactions between two nodes,* etc.*) without preparing a server for web services or programming clients. Usage of the interaction manager is shown in Fig. 6.1B in Additional file**1**.*

Similarly, the clustering algorithms implemented by the authors in MONGKIE (which are only really discussed in the Supplementary materials) just cover two algorithms that are already available in Cytoscape (through MCODE: http://baderlab.org/Software/MCODE [Bader and Hogue, 2003] and MCL: clusterMaker [Morris et al., 2011], which offers a whole range of clustering algorithms in addition). Not only is it not obvious whether the authors’ implementations bring any advantages compared to the widely used Cytoscape modules, the authors moreover present no validation of their own implementations.

**Authors’ response**: *We concede that the scope of clustering algorithms is rather limited because of the short development period. For user convenience, we implemented MCODE and MCL, two most frequently used algorithms. We confirmed the accuracy of implementation using test data with known answer. The source code for the unit test is available at**https://github.com/yjjang/mongkie/tree/master/ClusteringPlugins/test**. We would like to emphasize that the advantage of MONGKIE is not the diversity or superiority of clustering algorithms but efficient visualization and analysis of the resulting clusters in the subsequent steps with the group node function (see below).*

The authors also add extended support for group nodes - hyper-nodes containing multiple nodes - although the current text does not really make it clear what additional functionality the new tool provides beyond a new network graph layout and beyond what can be done with standard Cytoscape group nodes. I note that Cytoscape offers multiple graph layout algorithms and it’s not clear to me why the described algorithm would not be available or easily be made available as a plugin.

**Authors’ response**: *The group nodes in MONGKIE are very similar to the ‘compound nodes’ in Cytoscape that was just introduced in the recent release (version 3.3.0) during the review process of this paper. For their illustration of ‘compound nodes’, see the ‘Improved Group Visualization’ section at**http://www.cytoscape.org/release_notes_3_3_0.html**. Despite the conceptual similarity, MONGKIE provides some additional features in group visualization. For an example, the ‘compound nodes’ in Cytoscape can have only one shape (rounded rectangle) but MONGKIE supports various group shapes including rectangle, circle, and convex hull. Importantly, as described in the manuscript, we implemented the layout algorithm so that group nodes in MONGKIE are naturally laid out separately from others with minimum overlapping regions among groups. In addition, we note that the ‘compound nodes’ were implemented as a core feature (i.e. modification of core source code), not as a simple plugin. This suggests that the grouping function in MONGKIE would not have been possible as a Cytoscape plugin.*

The graphical user interface of MONGKIE further offers elaborate window and tab management, which the authors claim facilitates interactive analyses. The presented “User case study”, however, does not demonstrate the immediate benefits of these features. It is therefore not clear if the described system or article is a relevant contribution to research without major revision and extension of the original manuscript.

**Authors’ response**: *The user case study was just our attempt to illustrate representative features of MONGKIE and to convince users that MONGKIE is a viable and convenient option for network analysis and visualization. With the modification in the main text and extensive discussion in the review process, we feel that our manuscript has been substantially improved. We are extremely grateful to Dr. Kreil for helpful comments.*

**Recommendations**: The authors need to address a couple of open points - specifically: (1) The authors perform a first comparison of their novel software with Cytoscape, a powerful and long established alternative tool. It is not evident, however, to what degree their novel system introduces substantial new functionalities of direct interest to a user. This is critical because the adoption of a novel tool always comes with a considerable overhead and may well reduce the overall collaborative efficacy of the scientific community. The authors thus need to extend their article to explain to what degree any specific features are genuinely novel and of direct interest to others.

**Authors’ response**: *We have added description why we chose to develop a new program rather than a simple pluggin application. We agree that development of plugin is more desirable than development of an entirely new tool whenever possible. In our case, however, some functions such as group nodes, hyper-graphs, and usage of customized network as a background network model are only possible with modifying core engines of Cytoscape. We tried to minimize the learning overhead by adopting the same usage conventions as Cytoscape and by supporting import and export of networks in a compatible format with Cytoscape.*

(2) The authors perform a gene set enrichment analysis using only one method and report a known module retrieval rate of 40 % (2 modules out of 5 in agreement with literature). They conclude that “the result demonstrates that MONGKIE can be used to identify cancer driver genes and core gene modules effectively.” The authors either need to tone down their claims substantially, or provide a more meaningful benchmark. There is no point reporting measures of sensitivity without also reporting specificity. Also, the main question here really is to what degree their new tool facilitated or improved the analysis in comparison to alternative methods.

**Authors’ response**: *Measuring sensitivity and specificity is a delicate issue since it would depend on the input customized network and choice of clustering algorithms and parameters. Furthermore, there is no gold standard for true set of functional modules in GBM cancer biology. We just wanted to give an illustrative example of applying MONGKIE to analyze multi-omics data. Thus, we have added literature references for 2 modules in the text and provide relevant information for other modules as suggested by Prof. Yi. We have added description on merits of using MONGKIE for this type of multi-omics data analysis in the main text.*

**Minor issues**: Minor points to clarify or correct: (3) Please clarify to what degree, if any, customized networks in MONGKIE provide any novel functionality compare to Cytoscape [http://wiki.cytoscape.org/Cytoscape_User_Manual/Creating_Networks] and how that constitutes a substantial improvement.

**Authors’ response**: *We have described the difference in the response to summaries.*

(4) In the ‘User case study’ the authors apply various thresholds in the analysis - STRING score, p-value, and a particular correlation score - Pearson correlation, and apply a selected clustering algorithm - MCL. Although the focus of this paper is the introduction of a new integration / visualization tool, did the authors examine how changing the threshold and analysis algorithms influences results? And, more importantly, can they report how their tool helped in this investigation, a very typical application scenario? In particular, what role did its new features play in that?

**Authors’ response**: *As described in the response to (2) in Recommendations, MONGKIE offers a great flexibility in choosing background networks and clustering algorithms. The analysis procedure can be easily pipelined as well for routine analysis.*

**On revision:** In the revised manuscript, the authors have extended their comparison between customized network usage in Cytoscape and their system, sufficiently explaining the difference. They argue convincingly that working with customized graphs imported from web services in MONGKIE is simpler than in Cytoscape. In particular, there is no need for setting up a proxy server for this task. With some more effort, however, equivalent functionality can also be achieved in Cytoscape. The recently added implementation of group nodes (‘compound nodes’) in Cytoscape suggests that the second feature developed by the authors is also of more general interest. The authors now sufficiently compare their own implementation with the current Cytoscape version, validating the functionality of both as equivalent, with a minor difference of supporting a variety of group shapes. While this is valuable, I wonder if the parallel implementation of similar features could have been avoided by a more open development process in the community. The authors still claim that their system supports ‘many unique features’ although the main difference w.r.t. Cytoscape in the current implementation is the server-independent customized network support, some extended visualization options and a different user interface. Furthermore, while the authors argue that the development of novel clustering algorithms was not in the scope of the current publication, they do claim that "several network clustering algorithms are implemented" and that the system offers "flexibility in choosing (…) clustering algorithms", rather implying a multitude of available algorithms, whereas there currently are only two. Moreover, the text still implies that network-based integrative analysis was one particular method rather than a class of algorithms. In summary, while the authors address the comments made during original review, the software ‘MONGKIE’ introduced in this manuscript seems to have similar functionality to the latest version of Cytoscape. Its main benefits seem to be simpler setup, while it is missing the well established user community / selection of plug-ins of Cytoscape. Claims reaching beyond that are not sufficiently supported by the presented evidence, and the authors should either provide such material or tone down their claims.

**Authors’ response**: *We acknowledge that Cytoscape offers much greater flexibility in choosing clustering algorithms. We just want to emphasize that MONGKIE provides several unique features such as support of hyper-graphs, background network models, group nodes (compound nodes in the new release of Cytoscape) without losing valuable features of Cytoscape. I believe that availability of alternative solutions is desirable in scientific community and that users will choose the software according to their needs.*

## Additional file

Additional file 1: Supplementary text, figures, and data files. All text and materials were formated as a small self-contained website (1 HTML file with necessary figures and data files). Data files include input and result files of the case study including the fold change of expression values between tumor vs. normal conditions (in log_2_FC), average expression value of each gene in 4 GBM subtypes, GBM-altered subnetworks (nodes and edges) weighted by expression correlations between each pair of genes, and gene sets in 2 critical modules and their functional annotations. (ZIP 4315kb)

## Abbreviations

API: Application Programming Interface
GBM: Glioblastoma Multiforme
GO: Gene Ontology
JAX-RS: The Java API for RESTful Web Services
RCP: Rich Client Platform
REST: Representational State Transfer
SPI: Service Provider Interface
TCGA: The Cancer Genome Atlas
UI: User Interface

## References

[1] Gehlenborg N, O’Donoghue SI, Baliga NS, Goesmann A, Hibbs MA, Kitano H (2010). Visualization of omics data for systems biology. Nat Methods.

[2] Saraiya P, North C, Duca K (2005). Visualizing biological pathways: requirements analysis, systems evaluation and research agenda. Inf Vis.

[3] Saito R, Smoot ME, Ono K, Ruscheinski J, Wang P, Lotia S (2012). A travel guide to Cytoscape plug-ins. Nat Methods.

[4] McGuffin MJ, Jurisica I (2009). Interaction techniques for selecting and manipulating subgraphs in network visualizations. IEEE Trans Vis Comput Graph.

[5] Donoghue SIO, Gavin A, Gehlenborg N, Goodsell DS, Hériché J, Nielsen CB (2010). Visualizing biological data - now and in the future. Nat Publ Gr.

[6] Kincaid R, Kuchinsky A, Creech M (2008). VistaClara: an expression browser plug-in for Cytoscape. Bioinformatics.

[7] Brennan CWW, Verhaak RGWGW, McKenna A, Campos B, Noushmehr H, Salama SRR (2013). The Somatic Genomic Landscape of Glioblastoma. Cell.

[8] Szklarczyk D, Franceschini A, Wyder S, Forslund K, Heller D, Huerta-Cepas J (2015). STRING v10: protein-protein interaction networks, integrated over the tree of life. Nucleic Acids Res.

[9] Cerami E, Demir E, Schultz N, Taylor BS, Sander C (2010). Automated network analysis identifies core pathways in glioblastoma. PLoS One.

[10] van Dongen S. Graph clustering. Graph Stimul by flow Clust. 2000; PhD thesis: University of Utrecht.

[11] Furnari FB, Fenton T, Bachoo RM, Mukasa A, Stommel JM, Stegh A (2007). Malignant astrocytic glioma: genetics, biology, and paths to treatment. Genes Dev.

[12] The NetBeans Platform. https://netbeans.org/features/platform. Accessed 25 Sept 2015.

[13] Java API for RESTful Services (JAX-RS). https://jax-rs-spec.java.net/. Accessed 24 Nov 2015.

[14] Yu N, Seo J, Rho K, Jang Y, Park J, Kim WK (2012). hiPathDB: A human-integrated pathway database with facile visualization. Nucleic Acids Res.

[15] Aranda B, Blankenburg H, Kerrien S, Brinkman FSL, Ceol A, Chautard E (2011). PSICQUIC and PSISCORE: accessing and scoring molecular interactions. Nat Methods.

[16] Frick A, Sander G, Wang K (1999). Simulating Graphs As Physical Systems. Dr Dobb’s J Softw Tools.

[17] Resource for Biocomputing, Visualization, and Informatics (RBVI): Cytoscape Group Support. http://www.rbvi.ucsf.edu/cytoscape/groups/index.shtml. Accessed 24 Nov 2015.

[18] Holmes K, Roberts OL, Thomas AM, Cross MJ (2007). Vascular endothelial growth factor receptor-2: structure, function, intracellular signalling and therapeutic inhibition. Cell Signal.

[19] Holopainen T, Huang H, Chen C, Kyung EK, Zhang L, Zhou F (2009). Angiopoietin-1 overexpression modulates vascular endothelium to facilitate tumor cell dissemination and metastasis establishment. Cancer Res.

[20] Loffler H, Lukas J, Bartek J, Kramer A (2006). Structure meets function-Centrosomes, genome maintenance and the DNA damage response. Experimental Cell Research.

