# Supplementary material for: MONGKIE: an integrated tool for network analysis and visualization for multi-omics data
Source: Biol Direct. 2016 Mar 18;11:10. doi: 10.1186/s13062-016-0112-y (PMC4797132; doi:10.1186/s13062-016-0112-y)
Supplement: Additional file 1: — Supplementary text, figures, and data files. All text and materials were formated as a small self-contained website (1 HTML file with necessary figures and data files). Data files include input and result files of the case study including the fold change of expression values between tumor vs. normal conditions (in log2FC), average expression value of each gene in 4 GBM subtypes, GBM-altered subnetworks (nodes and edges) weighted by expression correlations between each pair of genes, and gene sets in 2 critical modules and their functional annotations. (ZIP 4315kb) [file 13062_2016_112_MOESM1_ESM.zip › indexR2.html]

  


MONGKIE 0.2.1 documentation


MONGKIE

Additional file 1

Table of Contents

- 1. Installation
- 2. Tutorial
- 3. Case Study
- 4. Network Visualization
- 5. Network Analysis
- 6. Interaction Sources
- 7. User Interface
- 8. Import and Export
- 9. Implementation
- 10. References

# MONGKIE

MONGKIE is a software platform for interactive visualization and analysis of complex omics data in the context of biological networks.

- All components for visualization (e.g. *Data-to-Visual mapping* and *Gene expression overlay*), network analysis of *defining subgroups*, and *functional interpretation* of network modules can be easily threaded into a pipeline that allows user interaction at each step.
- It was built on top of the *Plug-in architecture* to support application extension by third-party developers.

Important

In the *Case Study*, we demonstrate how MONGKIE can be used to identify driver gene candidates and core regulatory modules in the study of TCGA Glioblastoma Multiforme (Brennan et al., 2013).

## 1. Installation

This section describes how to install and run MONGKIE. Installation instructions are provided for `Linux` , `Mac OS X`, and `Windows`.

Caution

If you have an older version on your computer, you should uninstall it and remove the User Directories.

### 1.1. System Requirements

#### 1.1.1. Recommended hardware requirements

| OS | Processor | Memory | Disk space |
| --- | --- | --- | --- |
| Linux | Intel Core i5 or equivalent | 2GB (32-bit), 4GB (64-bit) | 1.5GB of free disk space |
| Windows | Intel Core i5 or equivalent | 2GB (32-bit), 4GB (64-bit) | 1.5GB of free disk space |
| OS X | Dual-Core Intel | 4GB | 1.5GB of free disk space |

#### 1.1.2. Java

MONGKIE is written in Java, and runs on the Java Runtime Environment. Therefore, the Java runtime (7 or 8) is required to install and run it. You can download the latest version of Java runtime from here for Windows and Linux, and OS X Lion (10.7), Mountain Lion (10.8), or Mavericks (10.9).

The tested Java versions are Java `8` and `7u67` for Windows, Linux, and OS X.

Caution

MONGKIE cannot be installed or run using Java 6.0, and OpenJDK is not supported, be sure to run the official Java version from Oracle’s website.

### 1.2. ZIP Distributions

1. Download the latest release of a ZIP distribution for your OS.
2. Unzip it to any directory on your system.
3. Run the executable file located in the `bin` directory

   > - On Linux, `mongkie/bin/mongkie`
   > - On Windows, `mongkie\bin\mongkie.exe`
   > - On OS X, `mongkie.app/Contents/MacOS/mongkie`

### 1.3. Installable Packages

Download the latest release of an installer for your OS.

#### 1.3.1. Linux and Windows

1. After the download completes, run the installer.

   > - For Windows, the installer file has the `.exe` extension. Double-click the file to run it.
   > - For Linux, the installer file has the `.sh` extension. You need to make the installer executable by using the following command: `chmod +x <installer-file>`. Type `./<installer-file>` to start the installation wizard.
2. Follow steps in the installation wizard.

#### 1.3.2. OS X

1. After the download completes, click on the downloaded `.dmg` file.
2. Drag the mongkie application in your Application folder.

### 1.4. User Directories

`userdir` is the directory where MONGKIE stores user configuration data such as window layouts, and various application options. Sometimes your `userdir` can be corrupted and this results in the MONGKIE behaving weirdly.

To fix such issues, delete `userdir` entirely, then restart MONGKIE, and allow it to generate a new `userdir` from scratch. In most cases, this should repair the problems.

`userdir` is located in:

- On Windows 2K/XP, `C:\Documents and Settings\<username>\Application Data\.mongkie`
- On Windows Vista or later, `C:\Users<username>\AppData\Roaming\.mongkie`
- On OS X, `/Users/<username>/Library/Application Support/mongkie`
- On Linux, `/home/<username>/.mongkie`

## 2. Tutorial

A step-by-step tutorial for the *Case Study* is available at our tutorial site online.

Note

Input data files to be imported into the MONGKIE in this tutorial were generated by the `extractAlteredNet_GBM.R`. Refer to *Case Study* for details about the method. Briefly, starting with somatic mutations, CNAs, and gene expression data sets downloaded from UCSC Cancer Browser, the `R script` does the following tasks.

- Calculate expression log2FCs of each gene in normal vs. tumor conditions.
- Calculate expression means of each gene in 4 GBM subtypes.
- Extract a sub-network of altered (somatic mutation or CNV) genes in STRING.
- Calculate expression correlations between each pair of genes in the extracted network.
- Write vertices, edges, and expression related data files.

You can download the R script and generated input files in `supplementary_data.zip`.

## 3. Case Study

High-throughput studies of tumor biology at multiple levels, including genome, transcriptome, and proteome, have been resulting in a greatly increased volume of cancer omics data. Given huge amount of cancer omics data, it is a major challenge to distinguish driver mutations from passengers, and to reveal functional relationships between them. One powerful approach to the challenge is to analyze data on the context of biological networks. For example, integration of mutation, copy number, and gene expression profiles with a biological interaction network has been proposed as an approach to identify functional cancer drivers, relying on the assumption that they will cluster on the network (Bertrand et al., 2015).

In this section, we demonstrate how MONGKIE can facilitate the study of structural pattern of altered genes in the TCGA study of Glioblastoma Multiforme (Brennan et al., 2013) on the STRING network to identify candidate driver genes and core gene modules perturbed by them.

Note

See *Tutorial* for a step-by-step tutorial for this case study.

### 3.1. Cancer omics data

Somatic mutations, DNA copy number alterations, and RNA-seq expressions level 3 data for TCGA GBM cases were obtained from the UCSC Cancer Browser. Downloaded files for each data type are listed in Table 3.1.

Table 3.1 TCGA GBM datasets processed by UCSC Cancer Browser


| Data Type | Downloaded Data File |
| --- | --- |
| Somatic Mutation | TCGA\_GBM\_mutation\_broad\_gene-2015-02-24.tgz |
| Copy Number | TCGA\_GBM\_gistic2thd-2015-02-24.tgz |
| RNAseq Expression | TCGA\_GBM\_exp\_HiSeqV2-2015-02-24.tgz |

Based on the 273 GBM cases with both somatic mutation and copy number information, each gene was considered altered if modified by a validated non-synonymous somatic nucleotide substitution, a homozygous deletion, or a multi-copy amplification. These somatic SNVs, indels, and called CNAs are combined to produce the gene-by-patient matrix M for gene alterations, where `M(i;j)` indicates whether the `gene i` is altered or not in the `patient j`, then an alteration frequency score for each gene was calculated by counting the number of patients in whom the gene is altered.

For gene-level expression profiles, we produced the gene-by-patient matrix G for gene expressions, where `G(i;j)` represents the expression level, which is a logarithmic scale of upper-quartile normalized RSEM (Li et al., 2011) estimats in tumor, for the `gene i` in the `patient j`.

### 3.2. Extraction of a GBM-altered network

We selected recurrently altered genes with somatic mutations in 6 or more patients, or CNAs in 9 or more patients from the alterations matrix (See above section). A total of 380 genes passed the frequency threshold. For each pair of those genes, we found all shortest paths in the STRING database (Confidence score > 900) with distance threshold 2, resulting in 175 altered genes and 815 linkers. To retain significant linkers only (Cerami et al., 2010), we applied the hyper-geometric distribution test for local enrichment against the global degree of each linker within the background network (See next section for details). After Benjamini & Hochberg (aka FDR) multiple testing correction (p-value < 0.01), we finally extracted a GBM-altered sub-network with `119 altered genes`, `72 linkers`, and `861 interactions` between them. The visualization of the extracted network in MONGKIE is shown in Fig. 3.1.

Fig. 3.1 GBM-altered network

Altered genes represented by `circles`, and linkers by `diamonds`; alteration frequencies were mapped to node sizes.

### 3.3. Statistical test for significant linkers

It is necessary to assess the probability that linker genes, which are not altered but extracted guilt by association, would connect to the observed number of altered genes by chance alone. The simplest and most widely used statistical test for such purpose is the `hyper-geometric distribution` test, where `successes in sample`: number of edges connecting the linker to altered genes in the extracted network (local degree), `successes in background`: global degree of the linker in the background network, `population size`: total number of genes in the background network, `sample size`: number of altered genes in the extracted network.

### 3.4. Network clustering

To give weights to the extracted network, we calculated Pearson Correlation Coefficients of expression levels in the expressions matrix (See above section) among all pair-wise interactions between genes in the extracted network, and then assigned the PCCs to weights of edges in the network. Next, we used a highly efficient network clustering algorithm, MCL (Van Dongen, 2000), to cluster the weighted network into a set of gene modules. The visualization of the result is shown in Fig. 3.2. Each module consists of gene set that are both topologically close in the PPI network, and highly correlated by expression abundance change in tumor conditions.

Fig. 3.2 Core gene modules in the GBM-altered network

Gene alteration frequencies in all GBM cases were mapped to node sizes; mean expression levels of each gene over cases of the *Mesenchymal* subtype were mapped to node colors; expression correlations in tumor cases were mapped to edge thicknesses. Two critical modules (See Results) are represented by different colors (one is `blue`, another is `red`; the others are `gray`)

### 3.5. Results

Two of the top 5 largest gene modules that are identified by network-based multi-omics (somatic mutations, copy number variations, and RNA expressions) analysis of TCGA GBM cases corresponded very closely to critical signaling pathways prior known to GBM biology. First one corresponded to the components of the `EGFR/PI3K signaling` pathway, including `EGFR`, `PDGFRA`, `PIK3CA`, and `PIK3R1` (see Fig. 3.3), and second one to the components of the `DNA damage response` and `Cell Cyle` including `TP53`, `CDKN2A/B`, `CDK4`, `MDM2/4` and `RB1` (see Fig. 3.4). Enrichment analysis of the two modules was performed using Enrichr, and the result is shown in Table 3.2.

Fig. 3.3 EGFR/PI3K signaling

Fig. 3.4 DNA damage response and Cell Cycle

Table 3.2 Gene list in 2 critical modules and their functional annotations in WikiPathways 2015


| Module | Gene List | Functional Annotation |
| --- | --- | --- |
| DNA damage response and Cell Cyle | `DDR_CellCycle.csv` | `DDR_CellCycle_WikiPathways.tsv` |
| EGFR/PI3K signaling | `EGFR_PI3K.csv` | `EGFR_PI3K_WikiPathways.tsv` |

In summary, we performed an integrated network analysis of multi-omics data to identify core network modules in the TCGA study of Glioblastoma Mutiforme, and the result revealed that our tool can be used to automatically identify cancer driver genes and core gene modules sharing structural pattern with those genes in a STRING network, thus to capture critical pathways that play important roles in tumor genesis.

## 4. Network Visualization

Like other network visualization tools, MONGKIE also provides basic graph representations (See *Visual representation of biological entities and interactions*) and many interactive ways to explore or edit a network, including Visual Editor UI, and Data-to-Visual Mapping (See *Visual editor UI and Data-to-Visual mapping*), zooming, filtering, searching (See *Exploring network*), and various graph layouts (See *Graph layouts*) etc. Furthermore, we implemented extended models for visualization of more complicated or interwoven biochemical reactions that can be multi-modal or hyper-graphs (Saraiya et al., 2005) involving more than two sub-components, therefore basic graph representations failed to model, for example, formation of protein complexes, interactions controlled by others (See *Pathway visualization*).

### 4.1. Visual representation of biological entities and interactions

Graphical representations of biological networks includes how to intuitively visualize a set of connected nodes (or vertice) corresponding to biological entities, including genes, gene products (protein, transcript factor, miRNA, etc.), small molecules (compound, metabolite etc.), protein family and complex, and their links (or edges), such as physical or genetic interactions, regulatory events (transcriptional and translational activation or inhibition, phosphorylation, etc.), co-expression, shared protein domain, complex formation, trans-location and other biochemical reactions.

Fig. 4.1 An example of visualization of biological networks

This illustrates how MONGKIE visually represents biological entities and relationships between them, by visualizing an example network, Glucocorticoid receptor regulatory network, which is a signaling pathway curated by the NCI-Nature PID (Schaefer et al., 2009).

MONGKIE provides the sophisticated data models for visualization of biological networks with advanced graph drawing techniques, and therefore can represent different types of biological entities and interactions between them with out-of-the-box visual styles, shown in Fig. 4.1. Both nodes and edges differ in their style according to their biological meaning. The style of nodes - e.g. label, font, shape, color, size and icon image - shows the type and state of biological components, and edges linking a relation participant with the information about the role also differ in their style - e.g. shape or thickness or color of lines, shape or color of arrows as well as label and font.

### 4.2. Pathway visualization

MONGKIE provides a built-in software module for pathway visualization, which supports visual analytics and exploratory studies of metabolic or signaling pathways in an interactive fashion tightly integrated with the human integrated pathway database, hiPathDB (Yu et al., 2012).

Like other types of biological interactions that can be modeled as regular binary-graphs, where interactions are between exactly two interactors, biological pathways also can be represented as a graph consisting of nodes and edges. Although these simple models have been shown to yield biological insights, biological pathways can be complex multi-modal or hyper-graphs (Saraiya et al., 2005), in which an edge could connect an arbitrary number of nodes or might connect a node to another edge. Therefore basic graph representations are incapable of modeling more complicated and interwoven biochemical reactions that involve more than two sub-components, e.g. the formation of protein complexes, and interactions that controlled by external controllers. In order to visually capture such complex biochemical events, MONGKIE presents an optimized pathway visualization based on the sophisticated visualization model taking into consideration these important domain-specific knowledges.

Fig. 4.2 Visual models for the pathway visualization

**A-1** Hierarchical modeling of protein complex assembly (orange border) can be flattened by splitting nodes representing complexes into their own individual members. **A-2** Protein complexes (red nodes). **A-3** Protein family (F icon). **A-4** Dimer (D icon). **A-5** Sub-cellular location (red icon). **B-1** Multiple edges (gray and blue). **B-2** Cellular states of nodes, mode of interactions of edges (activation, inhibition, phospholyration, etc.) **B-3** Self interactions. **B-4** Hyper-edges connect nodes to another edges (yellow), for example, one molecule (node) might prevent some other interaction (edge) from occurring.

Fig. 4.2 illustrates how diverse types of complex components and their relationships in biological pathways are visually represented in MONGKIE. A node representing a biological molecule in a pathway visualization may be either a protein, family, complex, dimer, enzyme, other small molecules (e.g. compound, metabolites), or especially a super-node which is a hierarchically decomposed composite node representing adjacency and inclusion relationships, e.g. the hierarchical modeling of protein complex assembly can be flattened by splitting nodes representing complexes into their own individual members (see Fig. 4.2 A). An edge in a pathway visualization represents a relationship or some form of interaction between nodes. The interaction could be one of many types: complex formation, activation, inhibition, aggregation, trans-location, catalysis, chemical modification, etc. In most cases, single-line connections are insufficient to capture the whole range of information contained in a biological pathway, because biological entities are often linked by more than one type of relationship. In such cases, multi-edge networks offer the possibility to link two entities by multiple edges, in which every edge having a different meaning. Also, hyper-edge connects a node to another edge, e.g. an inhibitory interaction (edge) actually indicates a biological process by which one molecule (node) might prevent some other interaction (edge) from occurring (see Fig. 4.2 B).

All theses different types of nodes and edges in the pathway visualization are visually represented with their distinct visual styles and additional informations, including their sub-cellular location, cellular state - e.g. activated, inhibited, phospholyrated, etc. Each component of pathways have informations about the originating data source, and hyper-links to the corresponding web pages is presented at the table view to link relevant databases - eg. NCBI, PubChem, etc. The force-directed layout algorithm (Frick et al., 1999) is optimized for the virtually automatic placement of components in the pathways.

### 4.3. Visual editor UI and Data-to-Visual mapping

Every component - nodes, edges, and groups - of the visualization model has a set of visual properties, including label text, text font, stroke, shape, size, color, line shape and width etc. These visual styles of them can be fully customized individually through the integrated Visual Editor UI which allows the user to edit them in any way the user desires with numerous predefined palettes. Actual UIs for editing visual properties of nodes, edges, and groups are shown in Fig. 4.3.

Fig. 4.3 Visual Editor UIs

Visual editor UIs for **(A)** nodes, **(B)** edges, and **(C)** groups with their members.

Each component in the network can have associated data attributes possibly describing visual properties of them. In addition to selective and manual editing of visual styles, MONKGIE also provides a very useful way to automatically set visual aspects of components based on their data attributes. This continuous or discrete Data-to-Visual attribute mapping allows researchers to synoptically view multiple types of data in a network context (see Fig. 4.4). Data-to-Visual mapping lets the user, for example, load omics data from various high-throughput experiments, e.g. expression profiles, and visually project them into the network by automatically transforming data to some graphical attributes, e.g. color, size, visibility etc.

Fig. 4.4 Continuous and discrete Data-to-Visual Mapping

Using an example network that are modeling cross-talks between biological pathways, **(A)** the number of genes in each pathway was continuously mapped to visual properties (size and color) of the corresponding pathway node. **(B)** data sources (KEGG or BioCarta) of each pathway were discretely mapped to node colors.

### 4.4. Exploring network

In order to substantially facilitate network navigation and information extraction, MONGKIE provides sophisticated options to explore networks in highly interactive ways, including `searching`, `filtering`, `grouping`, manual or automatic node `selection`, `highlight`, `dragging`, `zoom` and `panning` the display, `overview` of the complete network, and lastly `data table view` displaying attributes of nodes and edges in a tabular format.

MONGKIE provides easy-to-use `search functions` for the loaded network. One can enter any keyword or regular expression to search all data attributes held in the nodes and edges. The matching nodes or edges immediately highlighted in the visualization; and selection in the visualization propagates to the selection of the relevant rows in the data table scrolling to them, and vice versa. Furthermore, the network can be filtered down to interactions meeting a given `filtering constraint` according to their attributes. For instance, the network may be filtered to show only proteins occurring in particular locations, thus reducing the network complexity and restricting the one’s attention only to interactions within a given sub-cellular location, and this could greatly improve the visual perception of complex biological networks.

Given the large and complex networks, one common approach to interpret and visualize such networks is trying to display the complete network on the screen and providing functionalities to zoom, pan, and overview of the network for exploration. Like many other network visualization tools, MONGKIE provides these basic techniques for network navigation too. However, as the size and complexity of interactions grow, it is increasingly impossible to understand underlying structures and extract biological insights from such huge networks just using those basic navigation techniques. Another improved strategy is to dissect the complete network into smaller sub-networks that are manageable, biologically significant regions that can be understandable (Gehlenborg et al., 2010). These sub-networks are typically defined as, for example, sets of proteins that are occurring at the same sub-cellular location, or that belong to similar functional GO terms, or that are members of a densely connected cluster identified through the established network clustering methods. The resultant sub-networks are typically of a size that is more amenable to visualization and analysis.

In order to specifically support of these processes of dissection, MONGKIE provides a variety of ways to define groups of functionally or topologically related nodes, including enrichment analysis for functional modules (See *Over-representation analysis*), clustering analysis for topological clusters (See *Network clustering*), grouping by manual selection, or automatic partitioning of nodes according to their attributes. The defined groups or sub-networks are visually represented with distinct styles and importantly laid out separately from other parts in a way that automatically attracts each other nodes in a group while repelling other groups (See Fig. 5.1), and this geometric separation is essential to focus only on particular groups without being disturbed by unnecessary or noisy interactions. Additionally, one can create a new visualization of each sub-network, then analysis it independently from original one from which it derived.

MONGKIE is developed specially with these `grouping functionalities` in mind as one of main development goals so that it can be used to help dissect large interactions, thus reducing the overall complexity, and focusing on smaller but biologically interesting parts to gain biological insights without being overwhelmed by complexity and noises in the network.

### 4.5. Graph layouts

Graph layout algorithms are used to place graph nodes and edges in various geometric distribution for the clarity and readability of networks such that the number of edges crossing minimized and that the layout represents the overall structure of the network legibly.

A variety of state-of-the-art layout algorithms are implemented in MONGKIE, including Circular, Grid, Fruchterman-Reingold (Thomas et al., 1991), Radial Tree (Book et al., 2001), and Force-Directed (Frick et al., 19999) layouts, both for efficiency and quality (see Fig. 4.5).

Fig. 4.5 Graph Layouts

A variety of state-of-the-art layout algorithms are implemented in MONGKIE, including Circular, Grid, Fruchterman-Reingold, Radial Tree, and Force-Directed.

Like most of other typical network visualization softwares, we try a force-directed layout first because this layout can usually well organize most biological networks based on the non-deterministic algorithm that lets forces between nodes influence the position of the node in the network. All nodes exert repulsive force on the others whereas connected nodes are attracted to each other. After several iterations in which the positions are adjusted according to the calculated force, the layout stabilizes, keeping edge-crossings to a minimum (Herman et al., 2000 and Heer et al. 2005). It also visually animate the process for laying out the network so that one can watch nodes in the network incrementally being placed in optimum positions and can terminate the algorithm when a good layout is obtained. However, this layout quickly becomes inadequate if the size and complexity of network are too larger to handle and interact. For such cases, MONGKIE provides an opportunity to go without animations or to choose other faster but simpler one - e.g. circular, grid etc.

Fig. 4.6 UI for controlling layouts

Each layout algorithm can be easily started, canceled, and customized through the unified layout control UI shown in Fig. 4.6. Highly configurable layout algorithms also allow the user to change layout settings in real-time, and therefore dramatically increase user feedback and experience. For instance, settings of the force-directed layout, including gravity, spring and forces, can be configured and immediately applied even while the algorithm is running.

In addition to automatic layout algorithms, MONGKIE offers another way to interactively change the layout of the network by manually dragging each node or user-defined groups into any positions, and this is very useful in fine-tuning the automatic layout or emphasizing important nodes or biologically significant regions in the network by geometrically separating them from other parts.

## 5. Network Analysis

MONGKIE is designed for the both visualization of biological networks and also analysis of these networks with a seamless integration between two procedures. MONGKIE includes several of network analytical methods, such as network clustering, overlay of expression profiles, and over-representation analysis, to recognize putative functional or structural patterns, and uncover interesting biological meanings from biological interaction networks.

### 5.1. Network clustering

Fundamentally network-based approaches in systems biology are based on the hypothesis that biological entities rarely act alone in the cell, instead they interact spatiotemporally with others, forming modules, in order to perform specific cellular functions (Hartwell et al., 1999 and Alberts et al., 1998). Graph-based clustering algorithms that are mainly developed in graph theory and recently computational systems biology have been successfully applied to the study of detection of protein complexes (Krogan et al., 2006) or families (Enright et al., 2002), or identification of functions of uncharacterized proteins (Bader et al.,2002), or extraction of co-expressed clusters from co-expression networks (Lee et al., 2004), etc., and shown to obtain good performances for extracting such modules from a variety of biological interactions.

These so-called ‘network clustering’ methods are also known to be less susceptible to inherent false-positives and more accurately predict modules made up of functionally relate nodes rather than conventional iterative pair-wise clustering, where individual relationship - e.g. sequence similarity, co-expression - between two biological entities is investigated without considering structural patterns in their interactions with neighbors (Freeman et al., 2007).

MONGKIE currently incorporates two popular structure-based network clustering algorithms, including MCODE (Molecular COmplex DEtection algorithm), MCL (Markov CLustering algorithm), and these make it easy to find densely inter-connected, thus functionally related nodes in biological interactions, exploit both local and also global structural patterns, and visually map them onto the network. MCODE is a graph theoretic clustering algorithm for finding molecular complex in large protein interaction networks (Bader and Hogue, 2003). The MCODE plugin, which is implemented by porting from the pre-existing plug-in in Cytoscape (Shannon et al., 2003), identifies clusters by finding regions of significant local density. MCL is a fast and scalable unsupervised clustering algorithm for graphs based on simulation of the flow in the graph (Van Dongen, 2000). Because MCL is a robust state-of-the-art general purpose clustering algorithm for large graphs, it can be applied to any complex biological networks, e.g. protein functional relationship network to look for candidate cancer driver mutations and relevant functional modules they belong to (Wu et al., 2010).

Fig. 5.1 Network Clustering

This demonstrates the procedure of identification network clusters, organization them onto the network with distinct visual schemes, and how nodes in the same cluster are laid out coherently.

One can define resultant network clusters (or modules) as groups, therefore they, as described in *Exploring network*, can be visually organized and laid out onto the network with distinct visual schemes, as well as displayed in a tabular format. As shown in Fig. 5.1, each cluster and its members are visualized with a distinct color and shape according to their cluster membership, also laid out using the optimized force-directed layout algorithm that automatically attracts each member in a cluster while repelling other clusters. This helps users to visually interpret the coherence of clusters in the context of the network, that cannot be easily obtained by simply examining lists of clusters or their membership. Cluster nodes in the network can then be manipulated just like other general nodes for any exploratory purposes, e.g. one can select, then drag them in order to place in desired positions. One also can create a new visualization for a sub-network made up of nodes and edges in a cluster.

These features of network clustering and grouping in MONGKIE can facilitate to analyze or visualize the large data set of biological interactions in a more modular way that can provide biological insight into both local and also global structures in networks between biologically related nodes - e.g. a pathway affected by mutated genes, proteins belonging to a same complex or family, or cross-talk among biological pathways etc.

### 5.2. Gene expression overlay

Gene expression data obtained by micro-array experiments or RNA-seq techniques can provide powerful insights into underlying cellular states and dynamics when they are well integrated into the context of biological networks. Therefore, overlaying expression profile data onto a visualized network is an essential way in identifying a set of genes or proteins that share a related pattern of expression under a particular condition - eg. co-regulated gene sets and their interactions in a certain disease sample - or capture dynamic changes of their expression levels over a range of time points or different conditions (Gehlenborg et al., 2010). For these analyses, we require tools that allow users to visually represent expression profiles of the nodes in the network according to their expression level, thus better perceive the dynamic mechanism of a underlying biological system being guided by visualized expression patterns or changes.

MONGKIE provides powerful functionalities for visual analyses of high-throughput omics data in the context of networks, in particular for the gene expression data analysis of time series or multiple conditions. It supports dynamic visual representations, including a color gradient, size, and label of relevant nodes, that are easy to separate and interpret independently in order to depict the corresponding expression profile ratio of gene or protein nodes. Once expression profiles are imported as data attributes of nodes using the CSV file format, then a heat map visualization, which is used in a wide range of tools for the process of gene expression visualization (Gehlenborg et al., 2010) appears to display those expression data as a ratio-based graphical matrix, as well as incorporated within the graphical representation of nodes in the network (see Fig. 5.2).

An important challenge for expression data analysis is to interpret gene expression data produced from more than one condition, for example, time series experiments, or multiple perturbation studies. Therefore, it is necessary to consider all time points or conditions in order to detect temporal patterns and their changes in gene expression profiles whose values vary over time or different condition. This requires a selective or sequential visualization of multiple expression levels in the network context. MONGKIE allows users to incorporate such dynamics of gene expression profiles into the loaded network visualization by offering a way to change the visual mapping - e.g. color, size - of nodes to reflect the expression levels of a particular time point or condition according to the user selection.

Fig. 5.2 Overlaying of multiple expression profiles

This demonstrates the procedure of overlaying of gene expression data from multiple experiments onto genes in the network, and the UI for capturing dynamic changes of their expression levels over a range of different experiments.

Fig. 5.2 illustrates an example of this process, where gene expression profiles from multiple micro-array experiments in six cell lines were loaded into the heat map display, and also mapped on corresponding nodes in the network. A expression level of a particular experiment can be navigated using a sliding bar UI on the bottom of the window, and also the navigation process can be animated, as introduced in some tools (Hu et al., 2013 and Kincaid et al., 2008), by automatically switch to visualization of the next experiment with a predefined time interval. This is well suited to investigating by eye changes of expression levels within a group of interesting genes - e.g. genes that share a same functional term, members of a network cluster, or deferentially expressed genes - over given experiments. Furthermore, by arranging visualization windows in a grid (see *User Interface*), one can in parallel compare multiple visualizations of the same network, where each visualizes for its navigating experiment - known as the ‘small multiples’ approach (Gehlenborg et al., 2010).

### 5.3. Over-representation analysis

A group of interesting genes in the biological network, e.g. a module of potentially related genes has been found by the *Network clustering*, may be investigated to find biological pathways or other functional categories like GO (Gene Ontology Consortium, 2004) terms, where they are significantly over-represented. This approach, so-called ‘enrichment analysis’, is widely used in order to study the gene set for their over-representation in certain annotation classes that are usually related to biological functions of those genes (Huang et al., 2009).

Fig. 5.3 GO over-representation analysis

This demonstrates the procedure of enrichment analysis, and visual annotation of relevant nodes or regions (e.g. clusters) in the network with significantly over-represented GO terms.

MONGKIE provides a pipeline for this analysis, shown in Fig. 5.3, where researchers perform a statistical test for enrichment or depletion using the GO categories, in order to identify over-represented functional terms with statistically significance from the selected set of interesting genes in the certain network region. The result of analysis is displayed in table views with both a list of resultant functional terms and also statistics of the enrichment analysis, including number of enriched terms, number of population genes, number of query genes, and detailed information about each annotation term. In the result table, each row of resultant term has a background color that is mapped to its corresponding p-value - that was calculated using the hyper-geometric testing, and might be adjusted by one of following multiple testing correction methods: Bonferroni, Bonferroni-Holm, Benjamini-Hochberg, or Benjamini-Yekutieli - and the set of resultant term can be reduced based on user’s cut-offs and ranked by their statistical p-values.

Another optimized way to show the result of enriched functional terms in the hierarchical structure, e.g. Gene Ontology, is to display them in a tree table. In the tree table view, intermediate terms for tree hierarchy are automatically included, and users can expand or collapse any sub-tree in the view. Therefore it allows the user to interactively investigate terms according to specific biological complexity within the hierarchical structure as well as their significance.

Once a list of resultant term is displayed in a tabular format, users can select significant terms of interest, then visualize the group of relevant nodes or regions annotated with those terms in the network context by mapping distinguishable visual aspects to them. This visual mapping of functional terms in the context of the network has the distinct advantage of allowing the user to quickly identify by eye both biological functions of certain parts and also higher-order interactions between those parts in the network that would not be obvious without this type of visual representation.

## 6. Interaction Sources

Systems biology aims to study the relationships between molecular or functional components to gain insights into the underlying complexity and dynamics of biological processes from the properties or structure of the interactions (Kitano et al., 2002). This generally requires interaction data from diverse sources - e.g. protein-protein interactions, signaling or metabolic pathways - to be integrated, and analyzed together with data derived from high-throughput experiments, such as genomics, transcriptomics, and proteomics. One example of this integrative approach is to analyze altered genes in specific disease samples on the context of biological networks. By projecting the list of mutated, amplified, or deleted genes onto biological networks, one will find statistically significant subsets of related genes that are closely clustered as network modules or biological pathways affected by such genes.

Network-based multi-omics analyses can thus provide important insights into complex biological mechanisms and processes - e.g. the biology underlying disease etiology, or progression of several cancer types (Jones et al., 2008) - and reliable pathway databases as well as high-coverage protein interaction dataset are essential for such an analysis. MONGKIE is integrated with hiPathDB (Yu et al., 2012) which is the Human Integrated Pathway Database described below. Furthermore, it provides an interaction manager that allows users to incorporate external interaction data from multiple sources into the process of network analysis and visualization, and to manage them through an integrated UI.

### 6.1. hiPathDB

With heterogeneous biological pathway data sets in the diversity of potential data formats available, the integration of pathway resources has become an important issue in utilizing these resources systematically and efficiently. In order to utilize these resources to interpret and analyze genomic data using network-based analysis methods, the information stored in dispersed data repositories needs to be linked and combined in efficient ways, and strongly required to unify heterogeneous interactions in different pathway data sources into the one general network model.

Here, MONGKIE provides a built-in software module for highly interactive pathway visualization and exploration, tightly integrated with hiPathDB (Yu et al., 2012). hiPathDB is an integrated pathway database that combines curated human pathway databases of NCI-Nature PID (Schaefer et al., 2009), Reactome (Croft et al., 2010), BioCarta (Nishimura, 2001) and KEGG (Kanehisa and Goto, 2000). hiPathDB provides two different types of integration. One is the pathway-level integration - shown in Figure 3 of Yu et al., 2012 - which is conceptually a simple collection of individual pathways, was achieved by devising an elaborate model that takes distinct features of four databases into account, and subsequently reformatting all pathways in accordance with our model - shown in Figure 2 of Yu et al., 2012 - while maintaining molecular details of signaling processes. Another is the entity-level integration - shown in Figure 4 of Yu et al., 2012 - creates a single unified pathway, super-pathway, that encompasses all pathways by merging common components. Even though the detailed molecular-level information such as complex formation or post-translational modifications tends to be lost in the entity-level integration, such integration makes it possible to investigate signaling network over the entire pathways and allows identification of pathway cross-talks. Therefore, the unified super-pathway achieved by entity level integration facilitates the network-based analysis and navigation from the perspective of biological pathways.

### 6.2. Interaction Manager

In addition to biological pathways, there are other types of biological interactions that are well modeled by simple binary, pair-wise graphs, such as PPIs, TF-target, miRNA-target and genetic interactions, and they are also essential for the network analysis of genomic data. MONGKIE provides a elegant way to use these types of network as background networks for the exploratory network-based analysis and visualization, through the Interaction Manager UI, shown in Fig. 6.1 A.

Fig. 6.1 Interaction Manager

**(A)** Interaction Manager UI. **(B)** demonstrates the procedure of dynamic network construction starting with a small part of genes of interest (orange nodes).

Imported and managed interaction data sources can be utilized for various exploratory and analytical purposes, such as network generation from a scratch by search query, dynamic network exploration, expansion and filtering of interactions by their sources. For example, there are cases when the size of a interaction network is so huge that it is impossible to handle and visualize the complete network at once. It is recommended for such situations to follow the approach of a classical top-down exploration, in which rather than display the entire network in one display, initially start with a small part of nodes in the network, e.g. deferentially expressed genes, and then iteratively build a larger network by allowing the user to successively expand particular nodes of interest with their further interactions and neighbors.

The procedure for this strategy of top-down exploration provided by the Interaction Manager is shown in Fig. 6.1 B. It allows the user to expand interactions through the context-sensitive right-click menus on a selected nodes of interest (orange nodes), as well as just to connect existing nodes using the interaction dataset from a data source by selecting the check-box of that source in the interaction manager UI. Later, the user may delete neighbors out of the interest to reduce complexities of the network such that he/she focuses on the region of interest. Furthermore, each newly added node resulting from a expansion action is placed in an appropriate position with an animation, well incorporated into the force-directed layout algorithm (Frick et al., 1999, Heer et al., 2005), and this allows the user to easily preserve the so-called mental map (Misue et al., 1995) during exploration of the network.

Users can import their own binary interaction dataset into the interaction manager from files using standard formats - e.g. GraphML and CSV files. Additionally, visual styles of each interaction source can be fully customized through the Visual Style editor, and they will be persisted, the latter means that customized visual properties of edges from the interaction source as well as imported interaction dataset themselves is locally stored and will be available for styling edges on the next run of the application.

## 7. User Interface

Fig. 7.1 illustrates the main graphical user interface of MONGKIE. The main application window is made up of several dynamic views using the NetBeans window system that lets the user maximize and minimize, dock and undock, auto-hide and sliding, and drag-and-drop windows for well organizing views inside the main window.

Fig. 7.1 Graphical User Interface of MONGKIE

The Graphical User Interface (GUI) consist of a main visualization display with other windows, including analysis windows (network clustering, expression overlay, and enrichment analysis), visual editor, data-to-visual mapping window, data tables, statistics view, and an overview window.

The network visualization window is placed in the center with many context-sensitive menu items which allow users to easily communicate with other windows in an interactive way, access to currently important functionalities. Views in the left side of the main window includes GUIs for those functionalities that require user’s input or control actions, such as visual editor, data-to-visual mapping, network clustering, enrichment analysis, and graph layout. Those in the right side display a variety of contextual informations, including overview of the complete network, properties of selected nodes or edges, heat map visualization for gene expression data, and graphical charts that show various statistics in the visualization - e.g. groups or clusters in the network, node visibility after filtering etc. Those views that need to be organized in a tabular format together with search and filtering functionalities, such as the list of nodes or edges in the network, the result of enrichment analysis, are placed in the bottom of the main window.

While other views exists only once, the network visualization window can have multiple instances for different visualizations. This allows users to in parallel compare any number of visualized networks from different conditions, e.g. gene expression levels from multiple experiments (See *Gene expression overlay*), by tiling multiple visualization windows of the same network in a grid, where each one visualizes the information for its own condition.

Settings of the windows in the application, such as the size, position, and arrangement, are fully customizable by resizing or drag-and-drop. The flexibility of window management facilitates coherently working with multiple windows or views for the process of network visualization, navigation, or analysis. Also, these window settings are persisted across restart of the application, and later one can restore them to default settings.

Many parts of UI components and UX (User eXperiences) in MONGKIE are strongly inspired by Gephi (Bastian et al., 2009). We also use the Prefuse (Heer et al., 2005) java library for the graph data structure and interactive visualization.

## 8. Import and Export

Interaction data sets can be loaded and stored using different file formats, including GraphML (Graph Markup Language) and `CSV` (Comma-Separated Values). GraphML (Graph Markup Language) is a comprehensive and easy-to-use file format for graphs. Its main features include supports of storing directed or undirected or mixed graphs, hyper-graphs, hierarchical graphs, graphical representations, references to external data, and application-specific attributes. CSV (Comma-Separated Values) stores tabular data sets in a plain-text form, and is a basic file format that is widely supported by a wide range of scientific applications for loading or saving their data sets.

MONGKIE comes with built-in parsers for those files to read a graph or attributes of its nodes and edges from the given data file. GraphML file fully stores the structural information of a graph, hence it is quite straightforward to import a graph using that. CSV file, however, requires some extra steps to import a graph. As a first step, one need to prepare two files - one containing nodes and their attributes, and another containing an edge list and attributes. The CSV file containing nodes needs to include a column containing unique node IDs. The edge list CSV should include columns for `source` and `target`, containing node IDs of the start and end node for each edge. By simply going to `File -> Import from CSV Files`, a dialog window for importing both files will be appear, then the wizard UI will guide you to remaining steps. In following steps, one can make several modifications to properties of the importing graph - e.g. one can rename column names, specify if edges are `directed` or `undirected`, select columns of `source` and `target` for edges etc. Also, the type of each column in the node or edge table will be appropriately inferred according to the literal expression of their values - decimal data as `Integer`, floating points as `Double`, comma-separated text fields as `String Array`. When all steps are gone, the report dialog finally shows the summary of the imported graph, including number of nodes and edges, type of graph, issues occurred during the importing process etc. Furthermore, MONGKIE also use the CSV file as a input format in order to import expression profiles, or additional attributes of nodes or edges through the similar UI concepts applied to importing graphs.

MONGKIE is capable to export the graph in the visualization to 1) the GraphML file that contains all graph elements in one formatted file, or 2) two CSV files - each file stores the list of nodes and edges in a tabular format respectively. Exported graph serializations can be later used in not only MONGKIE itself, but also external graph visualization softwares (Shannon et al., 2003 and Bastian et al., 2009). Once satisfied with the network visualization, one can save it in one of multiple image formats - vector graphics, like SVG (Scalable Vector Graphics), EPS (Encapsulated PostScript), or bitmap images, like PNG, JPG, GIF, and BMP files.

MONGKIE also provides its own visual graph file format (`VLG`) that stores all visualization-wide properties - e.g. visual representations of each element, graph layout, node positions or visibilities, display scale, etc. - as well as data attributes of the whole graph in the network visualization, therefore one can save the current analysis in the `VLG` file, then later reopen it for continuing the analysis.

## 9. Implementation

MONGKIE is a desktop application written in Java 1.6+ based on the NetBeans RCP (Rich Client Platform), thus it is executable on all major operating systems such as Windows, Linux, or Mac, and provides robust ways to extend functionalities of the application with ease. In this section, we first describe the main software design and plug-in architecture focusing on its robustness and extensibility, then the multi-tiered system powered by RESTFul Web Service APIs for abstracting data and separating them from the business logics and presentation layers.

### 9.1. Plug-in architecture

MONGKIE is a java-based application built on top of the NetBeans Rich Client Platform that supports plug-in programming architecture, thus it is easy to implement various new plug-ins with additional functionalities. An overview of its modular architecture is given in Fig. 9.1.

Fig. 9.1 Schematic overview of the plug-in architecture implemented in MONGKIE

Based on its extensible architecture, MONGKIE provides core APIs (Application Programming Interfaces), SPIs (Service Provider Interfaces), and UI widgets for the base functionalities, such as graph visualization, network analysis, data integration, and many out-of-the-box supports that enable to build your own plug-ins onto the platform.

For example, The software module for *Graph layouts* provides well-defined APIs, SPIs and UI components that can be utilized by plug-in developers (See Fig. 9.2 A). Therefore, if you want to add a new layout algorithm into the MONGKIE, you only need to implement the logic of the layout algorithm without having to worry about other things like UI components, program states, window management, event handling, and data persistence and so on. All of these fundamental features for developing plug-ins are provided out of the box. This approach can allow a great deal of flexibility in the building various improvements of existing modules as well as the introducing of new functionalities or tools.

Fig. 9.2 Development and Management of Plug-ins

**(A)** an example of developing a layout plug-in implementing a new algorithm. **(B)** UI for plug-ins management.

MONGKIE also provides a GUI (Graphical User Interface) shown in Fig. 9.2 B, Plug-in Manager, in order to facilitate the management of different plug-ins. Hence, users can install, update, remove, activate, or deactivate individual plug-ins through the integrated UI, this allows the customization of the application functionalities according to their needs.

### 9.2. RESTFul Web service API

The multi-tiered system is applied for abstracting remote data sources and separating control logics from the data and presentation layers to improve data integrity and accessibility. We put retrieval logics for the remote data at the middle-tier, which enables to control database connections and provide unified and maintainable data access. RESTFul Web Service APIs, which are at the middle-tier, are implemented using the JAX-RS technology - the Java API for RESTful (Representational State Transfer) Web Services. Based on these web APIs, MONGKIE provides the functionality that allow users to access our integrated data sources and services from outside of the platform in a programmatic fashion through any REST clients, including Java Applet, Python or Ruby scripts (See Fig. 9.1).

Fig. 9.3 Overview of the 3-tier system implemented in hiPathDB (

**(1)** A REST client written for pathway visualization (blue colors), **(2)** RESTFul Web service at the middle tier (orange colors), **(3)** Relational database backend.

As an example, the implementation and usage of the hiPathDB (Yu et al., 2012) RESTful Web Service API is shown in Fig. 9.3. hiPathDB APIs allow researchers to retrieve data from hiPathDB database by offering methods to get pathways given their names, or get member genes given a list of pathways, and some others.

## 10. References

|  |  |
| --- | --- |
| [Albe98] | Alberts, B. (1998) **The cell as a collection of protein machines: preparing the next generation of molecular biologists.** *Cell* 92.3: 291-294. |

|  |  |
| --- | --- |
| [Bade02] | Bader, G.D., *et al*. (2002) **Analyzing yeast protein–protein interaction data obtained from different sources.** *Nature biotechnology*, 20.10: 991-997. |

|  |  |
| --- | --- |
| [BaHJ09] | Bastian, M., Heymann, S., & Jacomy, M. (2009) **Gephi: an open source software for exploring and manipulating networks.** *ICWSM*, 8, 361-362. |

|  |  |
| --- | --- |
| [BaHo03] | Bader, G.D. & Hogue, C.W. (2003) **An automated method for finding molecular complexes in large protein interaction networks.** *BMC Bioinformatics* 4(1), 2. |

|  |  |
| --- | --- |
| [Bert15] | Bertrand, D., *et al*. (2015) **Patient-specific driver gene prediction and risk assessment through integrated network analysis of cancer omics profiles.** *Nucleic acids research* gku1393. |

|  |  |
| --- | --- |
| [BoKe01] | Book, G., & Keshary, N. (2001) **Radial tree graph drawing algorithm for representing large hierarchies.** *University of Connecticut*. |

|  |  |
| --- | --- |
| [Bren13] | Brennan, C.W., *et al*. (2013) **The somatic genomic landscape of glioblastoma.** *Cell*, 155(2), 462-477. |

|  |  |
| --- | --- |
| [Cera10] | Cerami, E., *et al*. (2010) **Automated network analysis identifies core pathways in glioblastoma.** *PloS one*, 5(2), e8918. |

|  |  |
| --- | --- |
| [Crof10] | Croft, D., *et al*. (2010. **Reactome: a database of reactions, pathways and biological processes.** *Nucleic acids research*, gkq1018. |

|  |  |
| --- | --- |
| [Enri02] | Enright, A.J., *et al*. (2002) **An efficient algorithm for large-scale detection of protein families.** *Nucleic acids research*, 30.7: 1575-1584. |

|  |  |
| --- | --- |
| [Free07] | Freeman, T.C., *et al*. (2007) **Construction, visualisation, and clustering of transcription networks from microarray expression data.** *PLoS computational biology*, 3.10: e206. |

|  |  |
| --- | --- |
| [Fric99] | Frick, A., *et al*. (1999) **Simulating graphs as physical systems: a spring-embedder system for force-directed layout.** *Dr. Dobb’s Journal*. |

|  |  |
| --- | --- |
| [Gene04] | Gene Ontology Consortium. (2004) **The Gene Ontology (GO) database and informatics resource.** *Nucleic acids research*, 32.suppl 1: D258-D261. |

|  |  |
| --- | --- |
| [Gehl10] | Gehlenborg, N., *et al*. (2010) **Visualization of omics data for systems biology.** *Nature methods*, 7, S56-S68. |

|  |  |
| --- | --- |
| [Hart99] | Hartwell, L.H., *et al*. (1999) **From molecular to modular cell biology.** *Nature*, 402(6761 Suppl):C47-52. |

|  |  |
| --- | --- |
| [Heer05] | Heer, J., *et al*. (2005) **Prefuse: a toolkit for interactive information visualization.** In *Proceedings of the SIGCHI conference on Human factors in computing systems* (pp. 421-430). ACM. |

|  |  |
| --- | --- |
| [Herm00] | Herman, I., *et al*. (2000) **Graph visualization and navigation in information visualization: A survey.** *Visualization and Computer Graphics, IEEE Transactions on*, 6(1), 24-43. |

|  |  |
| --- | --- |
| [HuSL09] | Huang, D. W., Sherman, B. T., & Lempicki, R. A. (2009) **Bioinformatics enrichment tools: paths toward the comprehensive functional analysis of large gene lists.** *Nucleic acids research*, 37(1), 1-13. |

|  |  |
| --- | --- |
| [HuZC13] | Hu, Z., *et al*. (2013) **VisANT 4.0: Integrative network platform to connect genes, drugs, diseases and therapies.** *Nucleic acids research*, 41(W1), W225-W231. |

|  |  |
| --- | --- |
| [Jone08] | Jones, S., *et al*. (2008) **Core signaling pathways in human pancreatic cancers revealed by global genomic analyses.** *Science*, 321(5897), 1801-1806. |

|  |  |
| --- | --- |
| [KaGo00] | Kanehisa, M., & Goto, S. (2000) **KEGG: kyoto encyclopedia of genes and genomes.** *Nucleic acids research*, 28(1), 27-30. |

|  |  |
| --- | --- |
| [KiKC08] | Kincaid, R., Kuchinsky, A., & Creech, M. (2008) **VistaClara: an expression browser plug-in for Cytoscape.** *Bioinformatics*, 24(18), 2112-2114. |

|  |  |
| --- | --- |
| [Kita02] | Kitano, H. (2002) **Systems biology: a brief overview.** *Science*, 295(5560), 1662-1664. |

|  |  |
| --- | --- |
| [Krog06] | Krogan, N.J., *et al*. (2006) **Global landscape of protein complexes in the yeast Saccharomyces cerevisiae.** *Nature*, 440(7084), 637-643. |

|  |  |
| --- | --- |
| [LeeH04] | Lee, H.K., *et al*. (2004) **Coexpression analysis of human genes across many microarray data sets.** *Genome research*, 14.6: 1085-1094. |

|  |  |
| --- | --- |
| [LiDe11] | Li, B., and Dewey, C. N. (2011) **RSEM: accurate transcript quantification from RNA-Seq data with or without a reference genome.** *BMC bioinformatics*, 12(1), 323. |

|  |  |
| --- | --- |
| [Misu95] | Misue, K., *et al*. (1995) **Layout adjustment and the mental map.** *Journal of visual languages and computing*, 6(2), 183-210. |

|  |  |
| --- | --- |
| [Nish01] | Nishimura, D. (2001) **BioCarta.** *Biotech Software & Internet Report: The Computer Software Journal for Scient*, 2(3), 117-120. |

|  |  |
| --- | --- |
| [Sara05] | Saraiya, P., *et al*. (2005) **Visualizing biological pathways: requirements analysis, systems evaluation and research agenda.** *Info. Vis.*, 4(3), 191-205. |

|  |  |
| --- | --- |
| [Scha09] | Schaefer, C.F., *et al*. (2009) **PID: the Pathway Interaction Database.** *Nucleic Acids Res.*, Jan;37(Database issue):D674-9. |

|  |  |
| --- | --- |
| [Shan03] | Shannon, P., *et al*. (2003) **Cytoscape: a software environment for integrated models of biomolecular interaction networks.** *Genome research*, 13.11: 2498-2504. |

|  |  |
| --- | --- |
| [Thom91] | Thomas M.J., *et al*. (1991) **\*Graph Drawing by Force-directed Placement.** *Softw., Pract. Exper.* 21(11):1129-1164. |

|  |  |
| --- | --- |
| [VanD00] | Van Dongen, S.M. (2000) **Graph Clustering by Flow Simulation.** PhD thesis, University of Utrecht. |

|  |  |
| --- | --- |
| [WuFS10] | Wu, G., Feng, X., & Stein, L. (2010). **Research a human functional protein interaction network and its application to cancer data analysis.** *Genome Biol.*, 11, R53. |

|  |  |
| --- | --- |
| [YuSe12] | Yu, N., *et al*. (2012). **hiPathDB: a human-integrated pathway database with facile visualization.** *Nucleic acids research*, 40(D1), D797-D802. |

---

© Copyright 2015, Yeongjun Jang.
Revision `f085e032`.
