# Supplementary figures and images for: MONGKIE: an integrated tool for network analysis and visualization for multi-omics data
Source: Biol Direct. 2016 Mar 18;11:10. doi: 10.1186/s13062-016-0112-y (PMC4797132; doi:10.1186/s13062-016-0112-y)

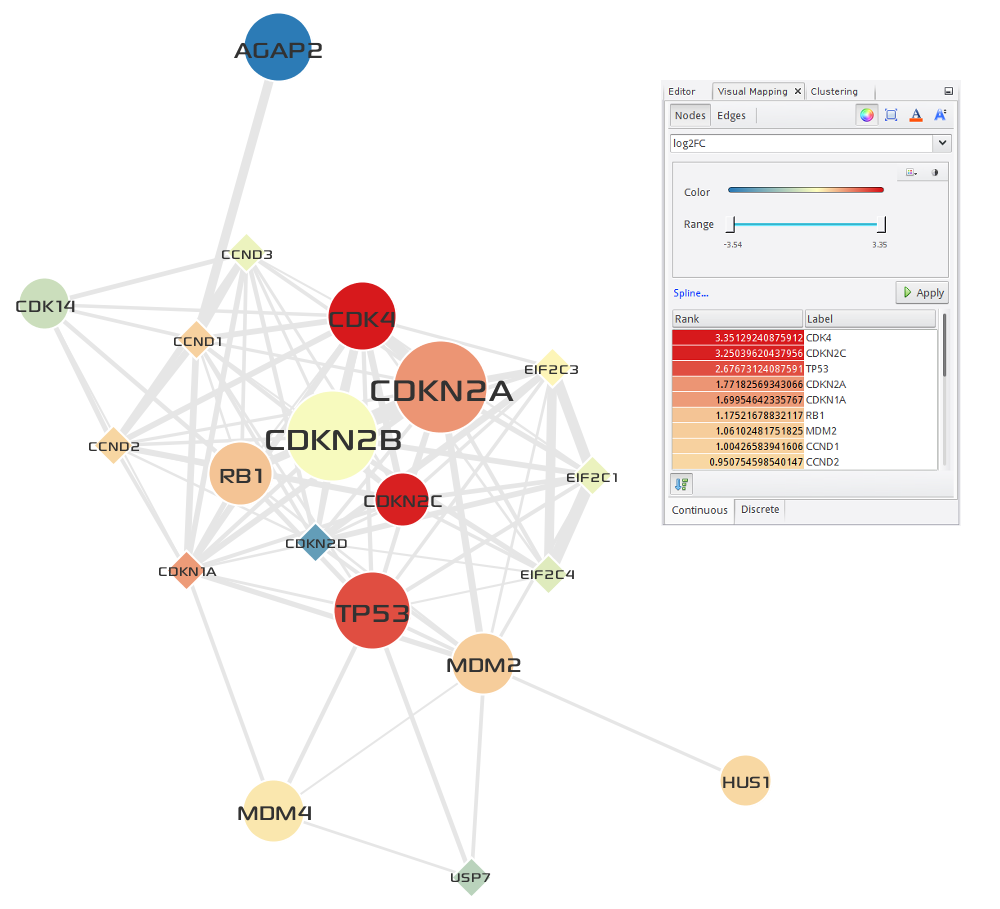

Supplement: Additional file 1: — Supplementary text, figures, and data files. All text and materials were formated as a small self-contained website (1 HTML file with necessary figures and data files). Data files include input and result files of the case study including the fold change of expression values between tumor vs. normal conditions (in log2FC), average expression value of each gene in 4 GBM subtypes, GBM-altered subnetworks (nodes and edges) weighted by expression correlations between each pair of genes, and gene sets in 2 critical modules and their functional annotations. (ZIP 4315kb) [file 13062_2016_112_MOESM1_ESM.zip › CellCycleR2.png]

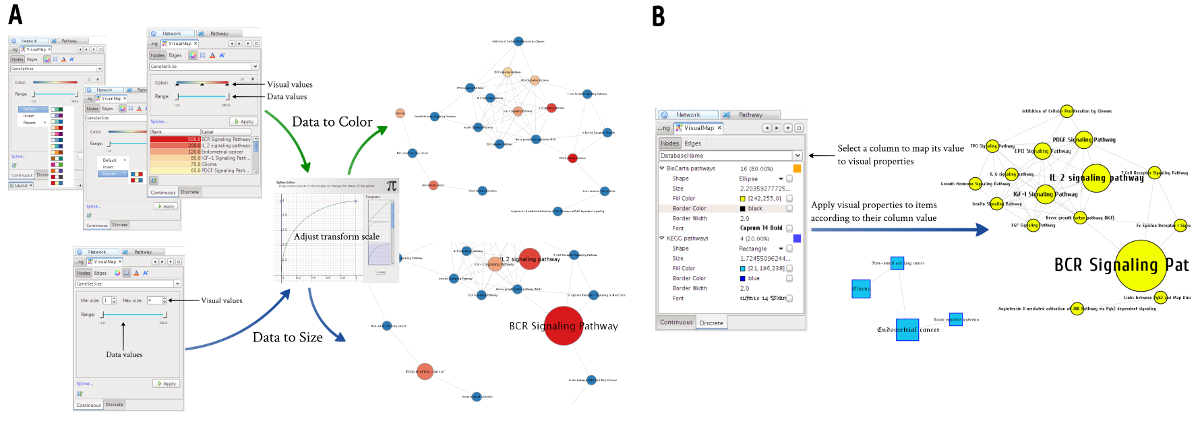

Supplement: Additional file 1: — Supplementary text, figures, and data files. All text and materials were formated as a small self-contained website (1 HTML file with necessary figures and data files). Data files include input and result files of the case study including the fold change of expression values between tumor vs. normal conditions (in log2FC), average expression value of each gene in 4 GBM subtypes, GBM-altered subnetworks (nodes and edges) weighted by expression correlations between each pair of genes, and gene sets in 2 critical modules and their functional annotations. (ZIP 4315kb) [file 13062_2016_112_MOESM1_ESM.zip › data_to_visual_mappingR2.png]

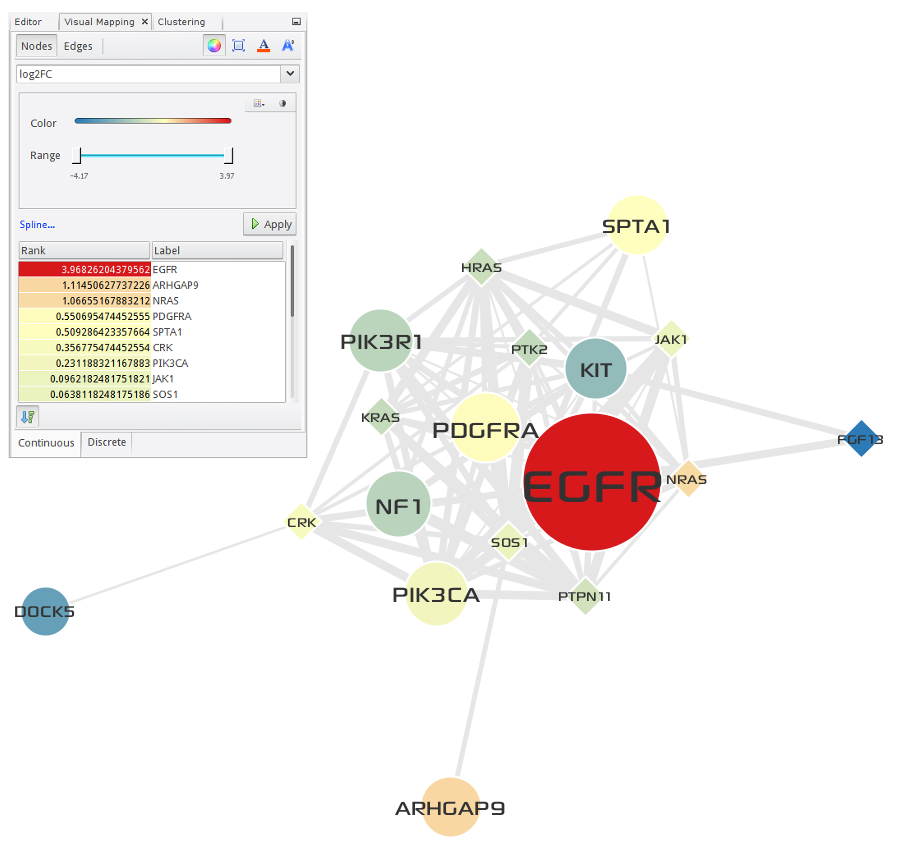

Supplement: Additional file 1: — Supplementary text, figures, and data files. All text and materials were formated as a small self-contained website (1 HTML file with necessary figures and data files). Data files include input and result files of the case study including the fold change of expression values between tumor vs. normal conditions (in log2FC), average expression value of each gene in 4 GBM subtypes, GBM-altered subnetworks (nodes and edges) weighted by expression correlations between each pair of genes, and gene sets in 2 critical modules and their functional annotations. (ZIP 4315kb) [file 13062_2016_112_MOESM1_ESM.zip › EGFRR2.png]

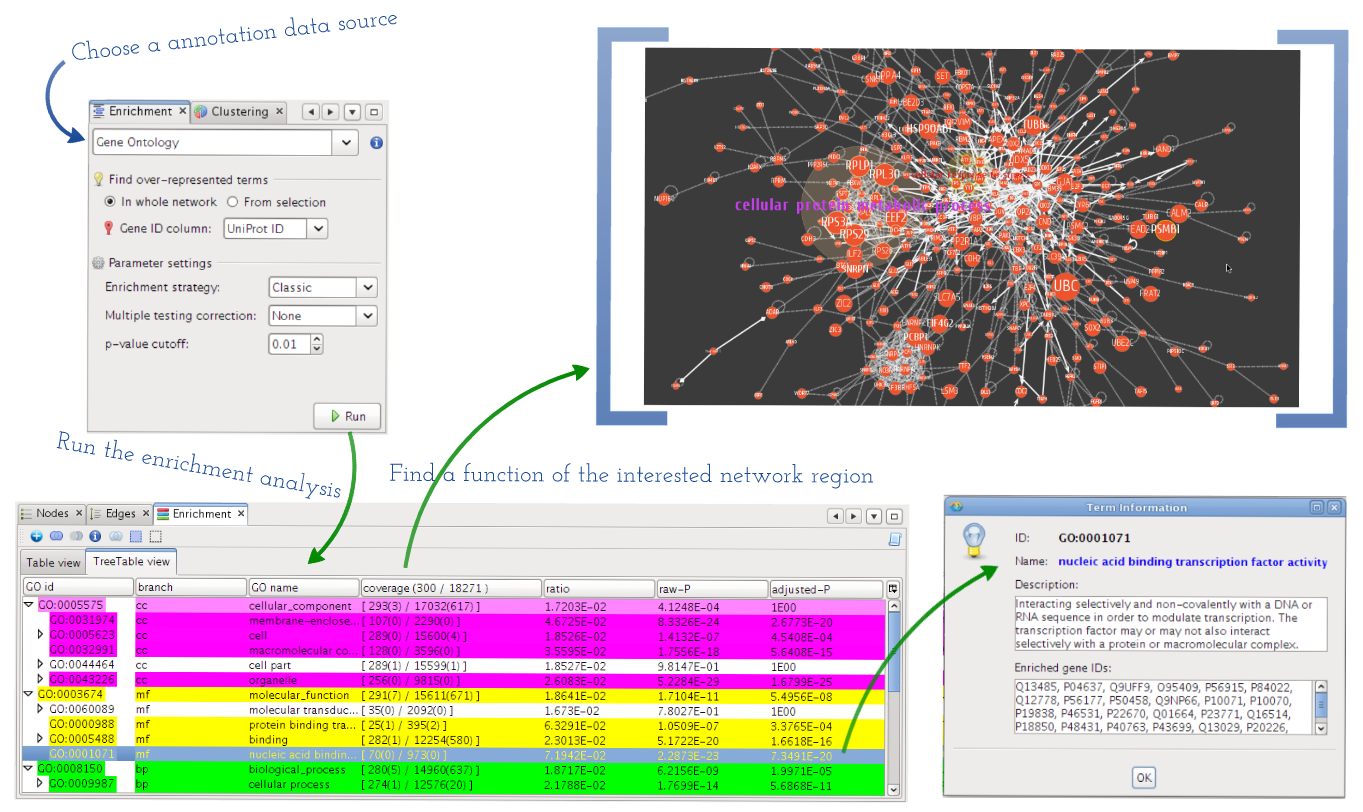

Supplement: Additional file 1: — Supplementary text, figures, and data files. All text and materials were formated as a small self-contained website (1 HTML file with necessary figures and data files). Data files include input and result files of the case study including the fold change of expression values between tumor vs. normal conditions (in log2FC), average expression value of each gene in 4 GBM subtypes, GBM-altered subnetworks (nodes and edges) weighted by expression correlations between each pair of genes, and gene sets in 2 critical modules and their functional annotations. (ZIP 4315kb) [file 13062_2016_112_MOESM1_ESM.zip › enrichment_analysisR2.png]

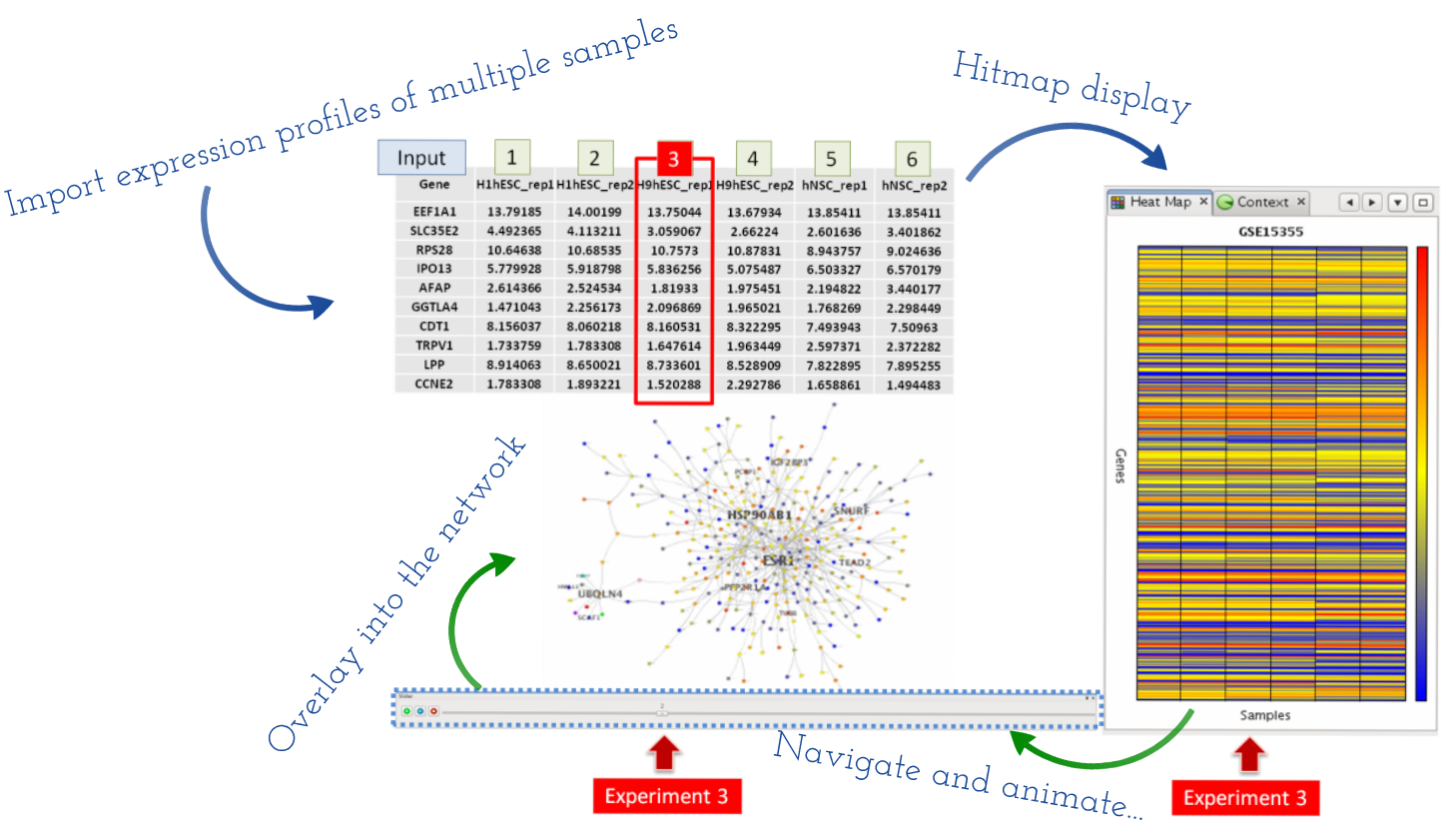

Supplement: Additional file 1: — Supplementary text, figures, and data files. All text and materials were formated as a small self-contained website (1 HTML file with necessary figures and data files). Data files include input and result files of the case study including the fold change of expression values between tumor vs. normal conditions (in log2FC), average expression value of each gene in 4 GBM subtypes, GBM-altered subnetworks (nodes and edges) weighted by expression correlations between each pair of genes, and gene sets in 2 critical modules and their functional annotations. (ZIP 4315kb) [file 13062_2016_112_MOESM1_ESM.zip › expression_overlayR2.png]

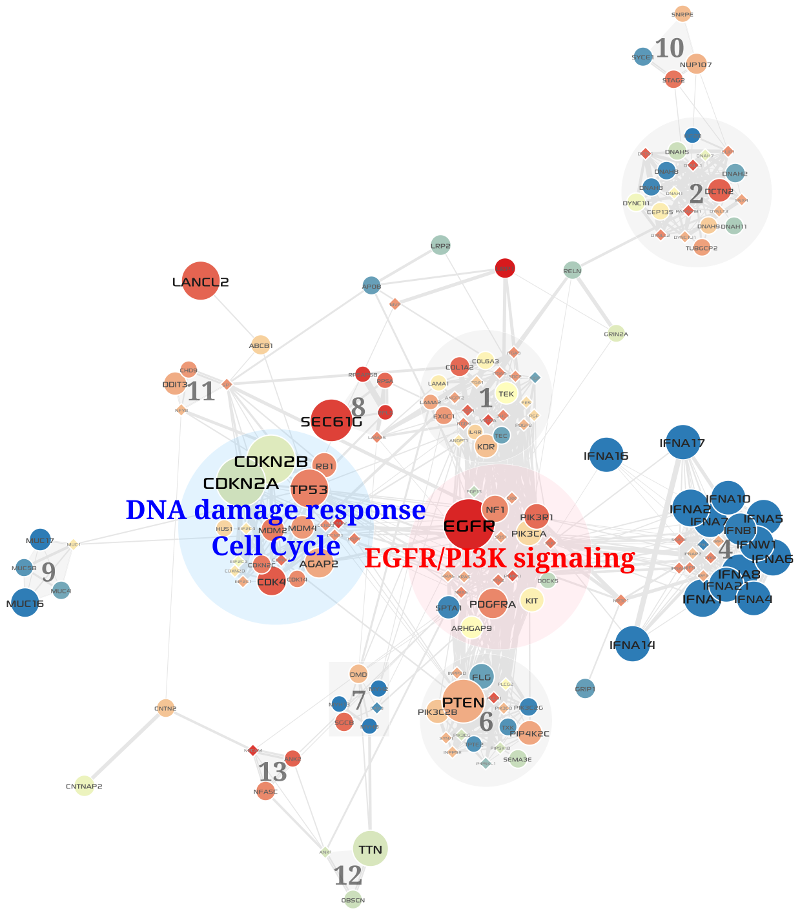

Supplement: Additional file 1: — Supplementary text, figures, and data files. All text and materials were formated as a small self-contained website (1 HTML file with necessary figures and data files). Data files include input and result files of the case study including the fold change of expression values between tumor vs. normal conditions (in log2FC), average expression value of each gene in 4 GBM subtypes, GBM-altered subnetworks (nodes and edges) weighted by expression correlations between each pair of genes, and gene sets in 2 critical modules and their functional annotations. (ZIP 4315kb) [file 13062_2016_112_MOESM1_ESM.zip › F1A_whole_networkR2.png]

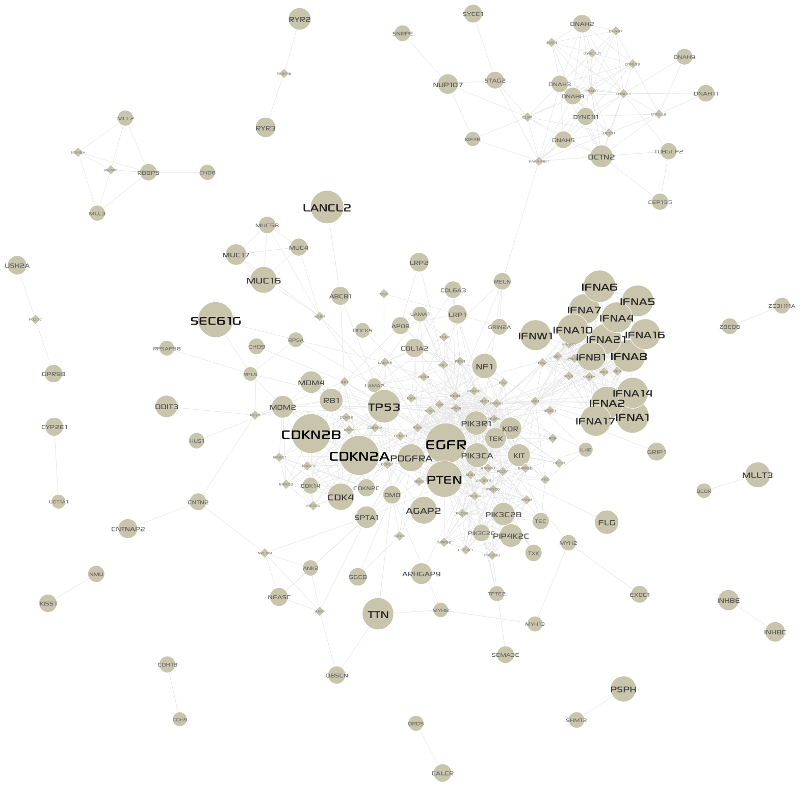

Supplement: Additional file 1: — Supplementary text, figures, and data files. All text and materials were formated as a small self-contained website (1 HTML file with necessary figures and data files). Data files include input and result files of the case study including the fold change of expression values between tumor vs. normal conditions (in log2FC), average expression value of each gene in 4 GBM subtypes, GBM-altered subnetworks (nodes and edges) weighted by expression correlations between each pair of genes, and gene sets in 2 critical modules and their functional annotations. (ZIP 4315kb) [file 13062_2016_112_MOESM1_ESM.zip › GBM_altered_networkR2.png]

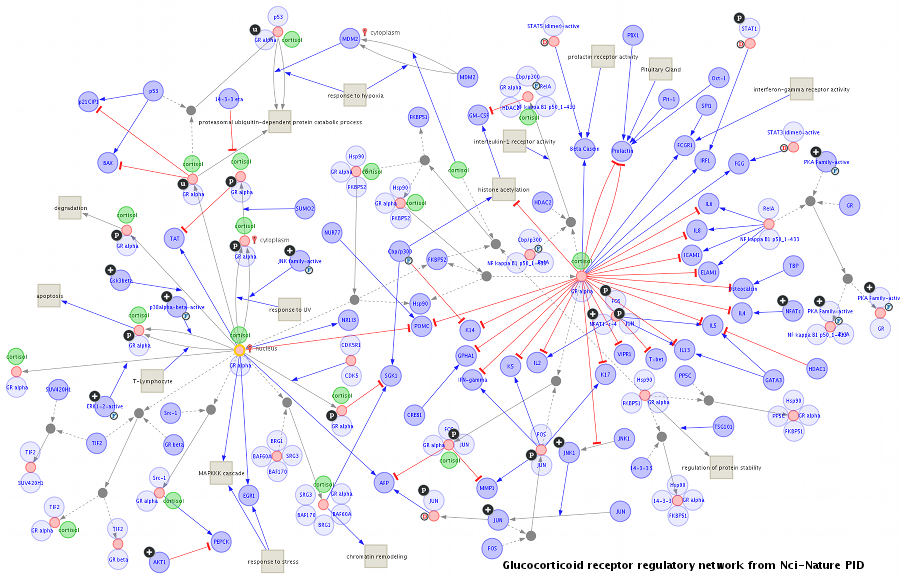

Supplement: Additional file 1: — Supplementary text, figures, and data files. All text and materials were formated as a small self-contained website (1 HTML file with necessary figures and data files). Data files include input and result files of the case study including the fold change of expression values between tumor vs. normal conditions (in log2FC), average expression value of each gene in 4 GBM subtypes, GBM-altered subnetworks (nodes and edges) weighted by expression correlations between each pair of genes, and gene sets in 2 critical modules and their functional annotations. (ZIP 4315kb) [file 13062_2016_112_MOESM1_ESM.zip › Glucocorticoid_receptor_regulatory_networkR2.png]

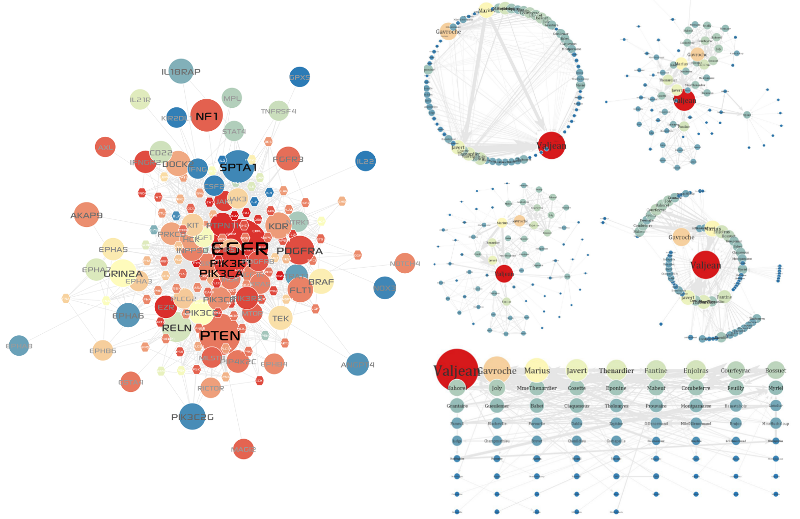

Supplement: Additional file 1: — Supplementary text, figures, and data files. All text and materials were formated as a small self-contained website (1 HTML file with necessary figures and data files). Data files include input and result files of the case study including the fold change of expression values between tumor vs. normal conditions (in log2FC), average expression value of each gene in 4 GBM subtypes, GBM-altered subnetworks (nodes and edges) weighted by expression correlations between each pair of genes, and gene sets in 2 critical modules and their functional annotations. (ZIP 4315kb) [file 13062_2016_112_MOESM1_ESM.zip › graph_layoutsR2.png]

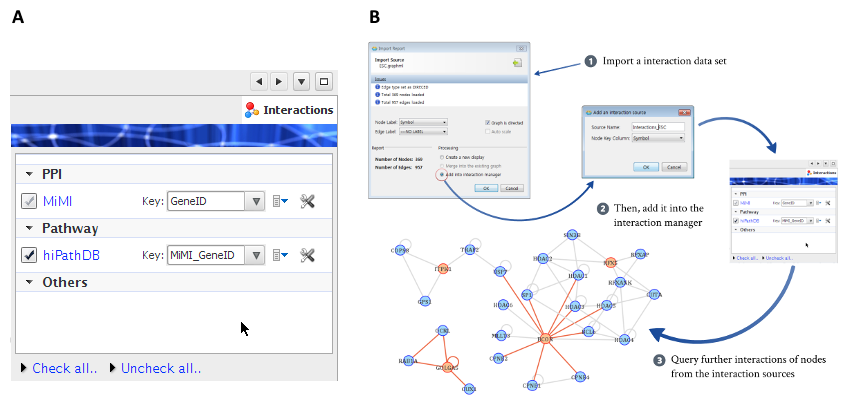

Supplement: Additional file 1: — Supplementary text, figures, and data files. All text and materials were formated as a small self-contained website (1 HTML file with necessary figures and data files). Data files include input and result files of the case study including the fold change of expression values between tumor vs. normal conditions (in log2FC), average expression value of each gene in 4 GBM subtypes, GBM-altered subnetworks (nodes and edges) weighted by expression correlations between each pair of genes, and gene sets in 2 critical modules and their functional annotations. (ZIP 4315kb) [file 13062_2016_112_MOESM1_ESM.zip › interaction_managerR2.png]

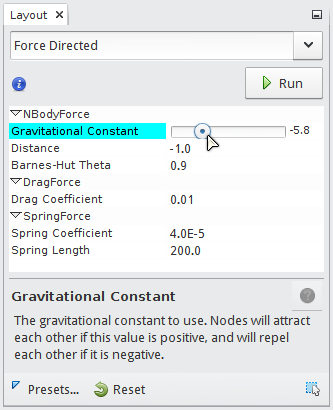

Supplement: Additional file 1: — Supplementary text, figures, and data files. All text and materials were formated as a small self-contained website (1 HTML file with necessary figures and data files). Data files include input and result files of the case study including the fold change of expression values between tumor vs. normal conditions (in log2FC), average expression value of each gene in 4 GBM subtypes, GBM-altered subnetworks (nodes and edges) weighted by expression correlations between each pair of genes, and gene sets in 2 critical modules and their functional annotations. (ZIP 4315kb) [file 13062_2016_112_MOESM1_ESM.zip › layout_controlR2.png]

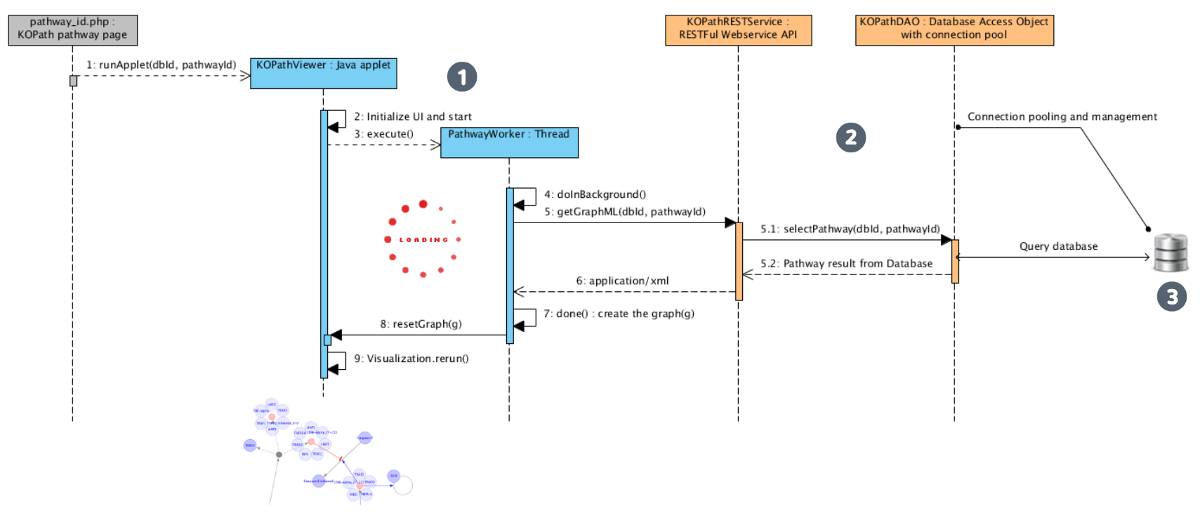

Supplement: Additional file 1: — Supplementary text, figures, and data files. All text and materials were formated as a small self-contained website (1 HTML file with necessary figures and data files). Data files include input and result files of the case study including the fold change of expression values between tumor vs. normal conditions (in log2FC), average expression value of each gene in 4 GBM subtypes, GBM-altered subnetworks (nodes and edges) weighted by expression correlations between each pair of genes, and gene sets in 2 critical modules and their functional annotations. (ZIP 4315kb) [file 13062_2016_112_MOESM1_ESM.zip › multi-tiersR2.png]

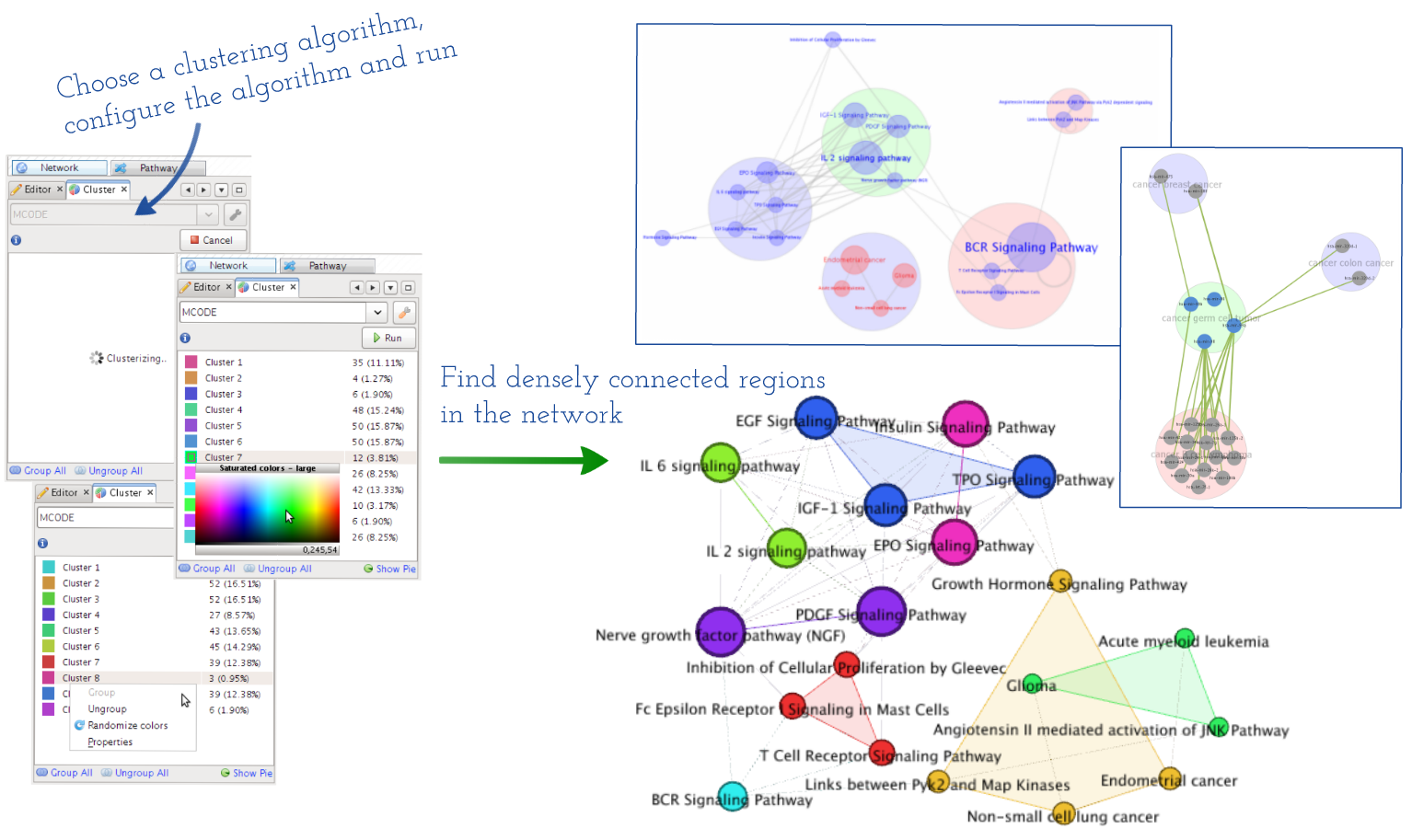

Supplement: Additional file 1: — Supplementary text, figures, and data files. All text and materials were formated as a small self-contained website (1 HTML file with necessary figures and data files). Data files include input and result files of the case study including the fold change of expression values between tumor vs. normal conditions (in log2FC), average expression value of each gene in 4 GBM subtypes, GBM-altered subnetworks (nodes and edges) weighted by expression correlations between each pair of genes, and gene sets in 2 critical modules and their functional annotations. (ZIP 4315kb) [file 13062_2016_112_MOESM1_ESM.zip › network_clusteringR2.png]

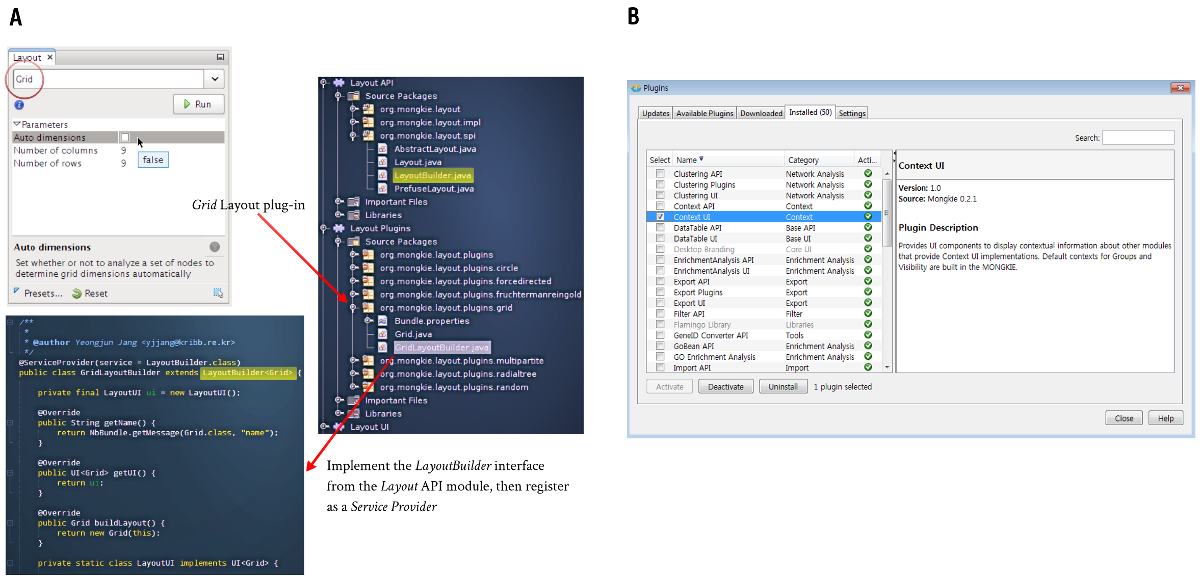

Supplement: Additional file 1: — Supplementary text, figures, and data files. All text and materials were formated as a small self-contained website (1 HTML file with necessary figures and data files). Data files include input and result files of the case study including the fold change of expression values between tumor vs. normal conditions (in log2FC), average expression value of each gene in 4 GBM subtypes, GBM-altered subnetworks (nodes and edges) weighted by expression correlations between each pair of genes, and gene sets in 2 critical modules and their functional annotations. (ZIP 4315kb) [file 13062_2016_112_MOESM1_ESM.zip › plugin_developmentR2.png]

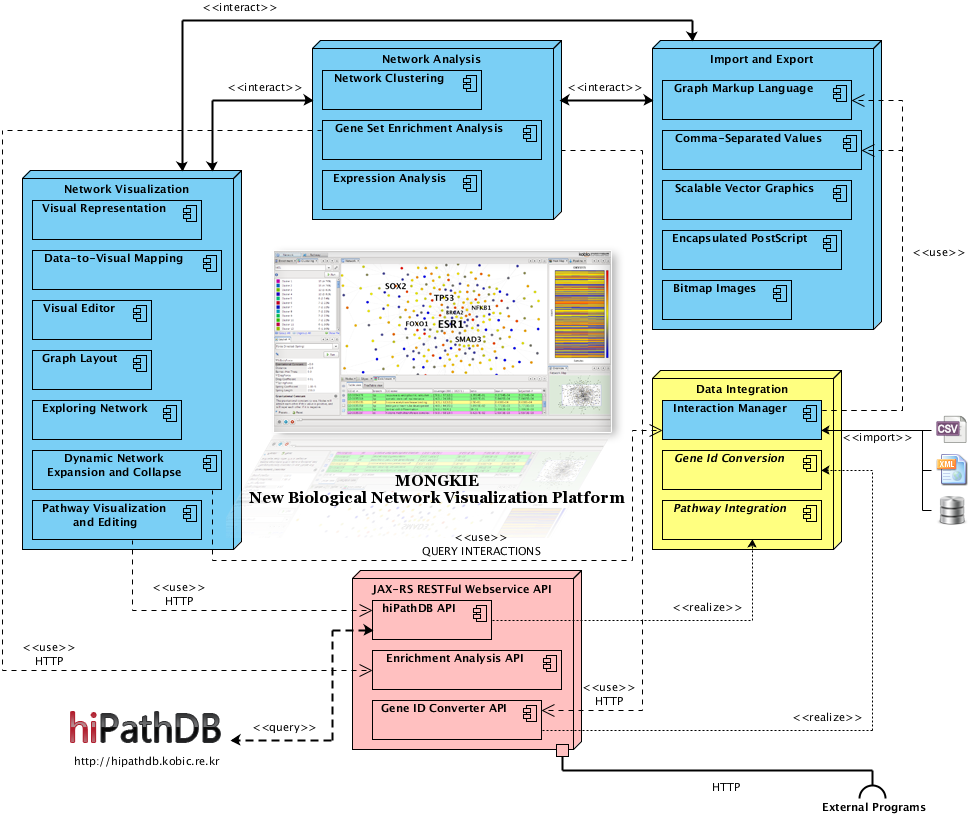

Supplement: Additional file 1: — Supplementary text, figures, and data files. All text and materials were formated as a small self-contained website (1 HTML file with necessary figures and data files). Data files include input and result files of the case study including the fold change of expression values between tumor vs. normal conditions (in log2FC), average expression value of each gene in 4 GBM subtypes, GBM-altered subnetworks (nodes and edges) weighted by expression correlations between each pair of genes, and gene sets in 2 critical modules and their functional annotations. (ZIP 4315kb) [file 13062_2016_112_MOESM1_ESM.zip › software_architectureR2.png]

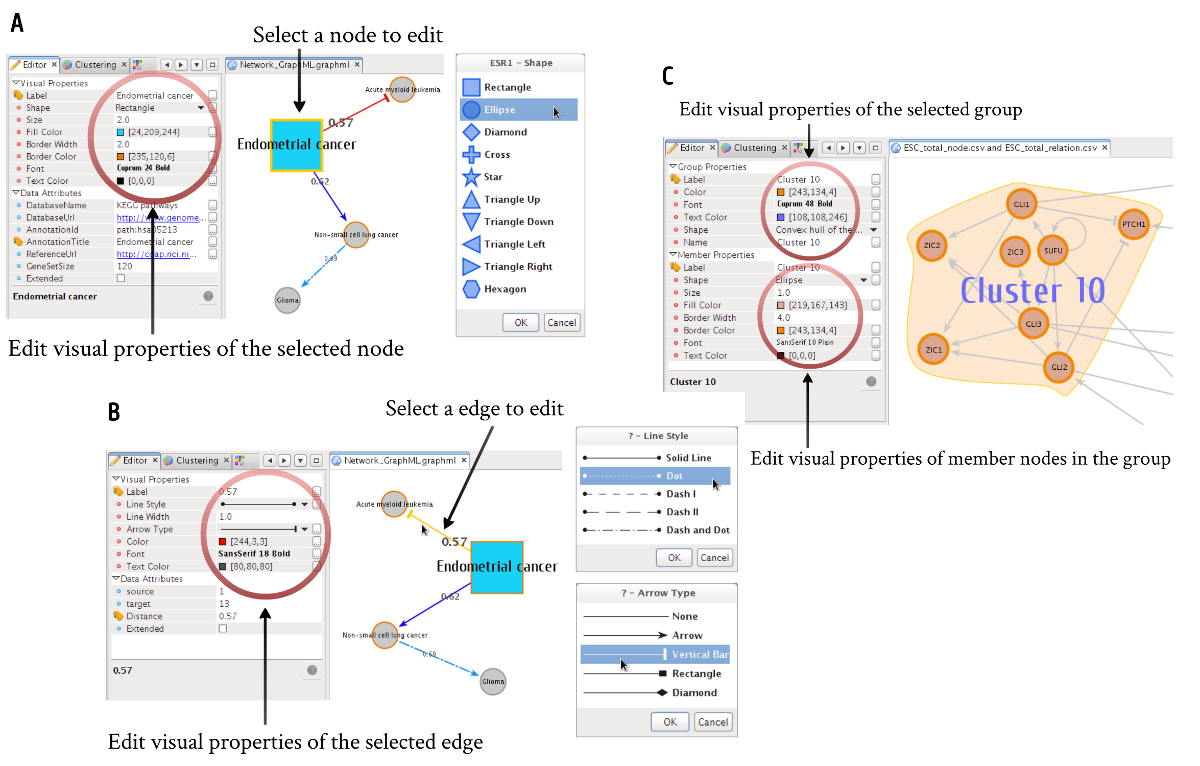

Supplement: Additional file 1: — Supplementary text, figures, and data files. All text and materials were formated as a small self-contained website (1 HTML file with necessary figures and data files). Data files include input and result files of the case study including the fold change of expression values between tumor vs. normal conditions (in log2FC), average expression value of each gene in 4 GBM subtypes, GBM-altered subnetworks (nodes and edges) weighted by expression correlations between each pair of genes, and gene sets in 2 critical modules and their functional annotations. (ZIP 4315kb) [file 13062_2016_112_MOESM1_ESM.zip › visual_editorsR2.png]

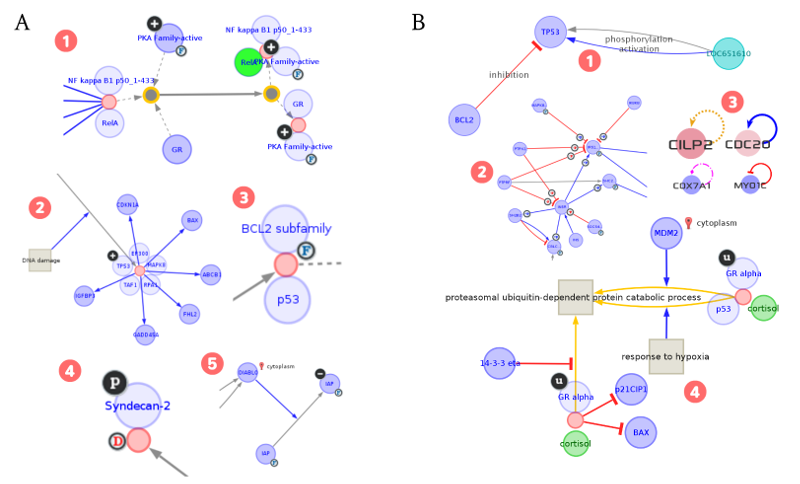

Supplement: Additional file 1: — Supplementary text, figures, and data files. All text and materials were formated as a small self-contained website (1 HTML file with necessary figures and data files). Data files include input and result files of the case study including the fold change of expression values between tumor vs. normal conditions (in log2FC), average expression value of each gene in 4 GBM subtypes, GBM-altered subnetworks (nodes and edges) weighted by expression correlations between each pair of genes, and gene sets in 2 critical modules and their functional annotations. (ZIP 4315kb) [file 13062_2016_112_MOESM1_ESM.zip › visual_modelsR2.png]
